# Characterization and Bioactive Potential of Secondary Metabolites Isolated from *Piper sarmentosum* Roxb.

**DOI:** 10.3390/ijms24021328

**Published:** 2023-01-10

**Authors:** Ismail Ware, Katrin Franke, Mthandazo Dube, Hesham Ali El Enshasy, Ludger A. Wessjohann

**Affiliations:** 1 Department of Bioorganic Chemistry, Leibniz Institute of Plant Biochemistry, 06120 Halle (Saale), Germany; 2 Institute of Bioproduct Development, Universiti Teknologi Malaysia (UTM), Johor Bahru 81310, Malaysia; 3 Institute of Biology/Geobotany and Botanical Garden, Martin Luther University Halle-Wittenberg, 06108 Halle, Germany; 4 German Centre for Integrative Biodiversity Research (iDiv) Halle-Jena-Leipzig, 04103 Leipzig, Germany; 5 City of Scientific Research and Technology Applications, New Borg Al Arab, Alexandria 21934, Egypt

**Keywords:** *Piper sarmentosum*, isolation, anthelmintic, antifungal, cytotoxic, antibacterial

## Abstract

*Piper sarmentosum* Roxb. (Piperaceae) is a traditional medicinal plant in South-East Asian countries. The chemical investigation of leaves from this species resulted in the isolation of three previously not described compounds, namely 4″-(3-hydroxy-3-methylglutaroyl)-2″-*β*-D-glucopyranosyl vitexin (**1**), kadukoside (**2**), and 6-*O*-*trans-p-*coumaroyl-D-glucono-1,4-lactone (**3**), together with 31 known compounds. Of these known compounds, 21 compounds were isolated for the first time from *P. sarmentosum*. The structures were established by 1D and 2D NMR techniques and HR-ESI-MS analyses. The compounds were evaluated for their anthelmintic (*Caenorhabditis elegans*), antifungal (*Botrytis cinerea*, *Septoria tritici* and *Phytophthora infestans*), antibacterial (*Aliivibrio fischeri*) and cytotoxic (PC-3 and HT-29 human cancer cells lines) activities. Methyl-3-(4-methoxyphenyl)propionate (**8**), isoasarone (**12**), and *trans*-asarone (**15**) demonstrated anthelmintic activity with IC_50_ values between 0.9 and 2.04 mM. Kadukoside (**2**) was most active against *S. tritici* with IC_50_ at 5.0 µM and also induced 94% inhibition of *P. infestans* growth at 125 µM. *Trans*-asarone (**15**), piperolactam A (**23**), and dehydroformouregine (**24**) displayed a dose-dependent effect against *B. cinerea* from 1.5 to 125 µM up to more than 80% inhibition. Paprazine (**19**), cepharadione A (**21**) and piperolactam A (**23**) inhibited bacterial growth by more than 85% at 100 µM. Only mild cytotoxic effects were observed.

## 1. Introduction

The Piperaceae family comprises numerous medicinal plants widely used in tropical and subtropical regions around the world. It consists of five genera namely *Verhuellia, Zippelia, Manekia, Piper* and *Peperomia* [1]. The most frequently described genera are *Piper* and *Peperomia* [1,2,3]. The *Piper* genus contains about 1000–2000 species with dominant species in their native habitat [4]. Many species of *Piper* have been used as traditional medicine to treat toothache, fever, chest, pain, cough, asthma, etc. [2]. Previous phytochemicals studies of the *Piper* genus resulted in the isolation of amide alkaloids, lignans, neolignans and phenylpropanoids as major constituents [2,5]. These isolated compounds displayed a wide range of biological effects including antifungal, antitumor, anti-inflammatory and antioxidant activities [2,6,7,8].

*Piper sarmentosum* Roxb. (Piperaceae) is a creeping plant whose vernacular name varies from country to country. It is known as *Kaduk* and *Pokok Kadok* in Malaysia, *Chaplu* in Thailand, *Sirih duduk, Akar buguor* or *Mengkadak* in Indonesia, *Bolalot* in Vietnam and *Jiaju, Gelou, Jialou* and *Shanlou* in China [9,10,11]. This species is widely distributed in tropical regions in Northeast India, Southeast Asia and parts of China and has been commonly used in traditional medicine and also as food flavoring agents [12,13]. In Malaysia, the plant is also eaten raw as vegetable and the leaves are boiled in water and taken to relieve fever in malaria and treat coughs, flu, and rheumatism [14]. Furthermore, the whole plant, roots, leaves and fruits of *P. sarmentosum* have been used for the treatment of colds, gastritis, rheumatoid joint pain, abdominal pain, toothache, diabetes mellitus, worm infections and other diseases for many decades [15,16].

Modern pharmacological studies have shown that crude extracts of *P. sarmentosum* possess a wide range of biological activities such as antibacterial [17], anti-fungal [18], anti-osteoporosis [19,20], anti-depression and neuroprotective [21,22], anti-inflammatory [23,24], anti-cancer [25], hypoglycemic [26], insecticidal [27,28], and antihypertensive activities [29,30]. A variety of chemical constituents, including essential oils, alkaloids, flavonoids, lignans and steroids, have been isolated mostly from the leaves and aerial parts of *P. sarmentosum* [15]. However, although a large number of chemical components have been isolated and identified from this species, only a few pure compounds have been studied with respect to their biological activity.

In the present study, we report the isolation, structure elucidation and biological effects of the previously undescribed compounds **1**–**3** along with 31 known compounds from the methanolic leaf extract of *P. sarmentosum*.

## 2. Results and Discussion

### 2.1. Isolation and Structure Elucidation

**Figure 1:**
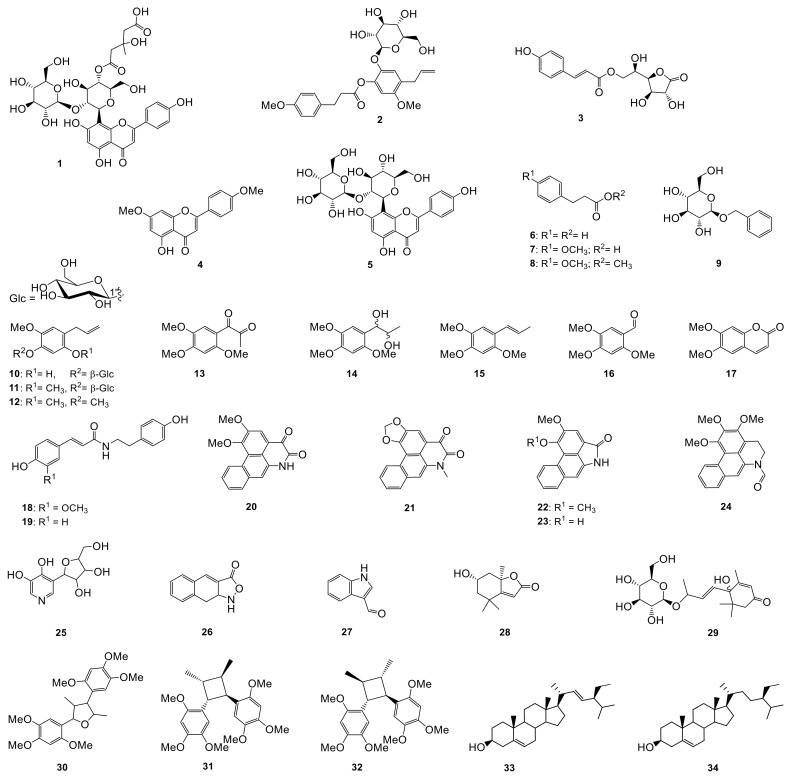
Chemical structures of compounds **1**–**34**.

The dried leaves of *P. sarmentosum* were extracted with 80% of aqueous methanol. The crude extract was suspended in H_2_O and successively divided by liquid-liquid partition between water and *n*-hexane, ethyl acetate, and *n*-butanol. The constituents from the three organic fractions were purified by repeated column chromatography on silica gel, Sephadex LH20, RP and Diaion HP20 and semi-preparative HPLC, which yielded three hitherto undescribed compounds (**1**–**3**) together with 31 known compounds (Figure 1). The known compounds include two flavonoids (**4**, **5**), twelve phenolics (**6**–**17**), two amides (**18**, **19**), eight alkaloids (**20**–**27**), two terpenes (**28**, **29**), three lignans (**30**–**32**), and a mixture of sterols (**33**, **34**) which were identified by spectroscopic analysis and comparison of the data obtained with literature values.

Compound **1** was isolated as brownish liquid and has a molecular formula of C_33_H_38_O_19_, which was deduced from the (-) HR-ESI-MS [M-H]^-^ ion at *m/z* 737.1937, calcd. for C_33_H_37_O_19_ 737.1935. The ^1^H-NMR and ^13^C-NMR data (Table 1, Figures S1 and S2) indicate an olefinic signal at δ_H_ 6.62/δ_C_ 103.6 which was typical of a H-3 flavone signal, an aromatic signal at δ_H_ 6.24/δ_C_ 99.4 that confirms the pentasubstituted status of ring A, two doublets at δ_H_ 8.04 (2H, d, *J* = 8.7 Hz, H-2′/6′) and δ_H_ 7.03 (2H, d, *J* = 8.7 Hz, H-3′/5′), typical of a A_2_B_2_ spin system on the ring B. Altogether, these data determined the aglycone component of **1** as apigenin structure [31]. The NMR data revealed a C-8 (or C-6)- substituted flavone comprising two anomeric sites resonating at (δ_H_ 5.09/δ_C_ 73.8) and at (δ_H_ 4.26/δ_C_ 105.3) that were diagnostic of their *C*-glycosylated and *O*-glycosylated status, respectively. The other signals between δ_H_ 2.76 and 5.26 can be assumed to be caused by the protons of the sugar moieties. In addition, high field signals at δ_H_ 2.72, 2.73, 2.83 and 1.46 indicate the presence of further substituents.

**Figure 2:**
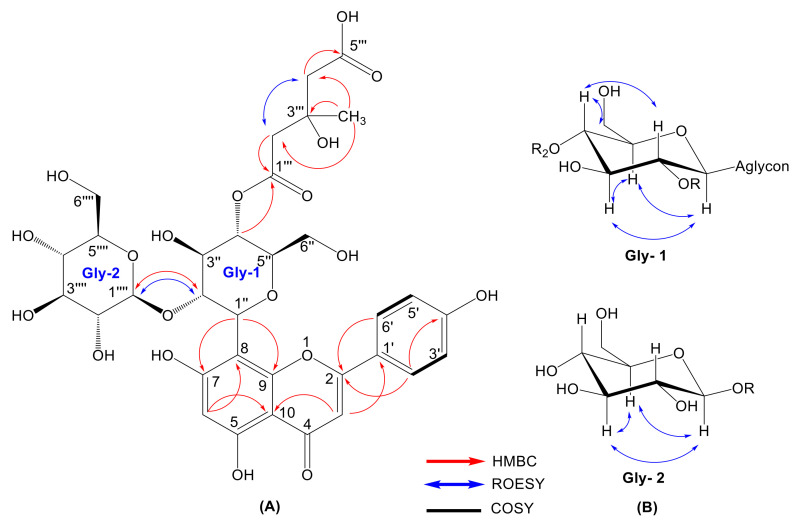
Details of NMR characteristics of compound **1**. (**A**) Full structure; (**B**) Detail of ROESY correlation of glucose moieties.

^1^H and ^13^C signals of **1** were further assigned by extensive analysis of HSQC, ^1^H-^1^H COSY and HMBC spectra (Figure 2, Figures S3–S6). HMBC experiments show correlation between the anomeric proton δ_H_ 5.09 (H-1″) and apigenin C-7/9 confirming that the disaccharide is bound by a *C*-glycosidic linkage at C-8. A further HMBC correlation from the anomeric proton δ_H_ 4.26 (H-1″″) to C-2″ (δ_C_ 80.7) revealed the interglycosidic linkage (1→2). The large coupling constant of the anomeric protons (*J*_H-1″-H-2″_ = 10.1 Hz and *J*_H-1″″-H-2″″_ = 7.7 Hz) determined the *β*–configuration of both sugar moieties. The spectral data of **1** show similarity to those of 2″-glucosylvitexin (**5**) [32], with some differences at C-4″ of the gly-1 unit. The downfield chemicals shift of the gly-1 methin proton (Table 1) suggests acylation of the gly-1 unit at C-4. This proton signal is correlated in the HMBC spectrum with a carboxylic carbon at δ_C_ 172.2. The HSQC and HMBC spectra show this carboxyl to belong to an aliphatic acyl moiety, which is successively linked to a methylene group [δ_C_ 46.2; δ_H_ 2.73; 2.83], a central quaternary carbon (δ_C_ 70.9), another methylene group [δ_C_ 46.4; δ_H_ 2.72; 2.83], and a terminal carboxyl residue (δ_C_ 174.8). Additionally, the central quaternary carbon (C-3‴) shows a HMBC correlation with a methyl group at δ_H_ 1.46 (3H, s) and δ_C_ 27.7. Consequently, the aliphatic acyl moiety was identified as a 3-hydroxy-3-methylglutaroyl (HMG) substituent. Due to the low obtained amount of compound **1** (2 mg), the structure elucidation had to rely on non-destructive methods and thus the absolute configuration of the HMG substituent at C-3‴ could not be determined. Compound **1** showed negative optical rotation of ${\left[\alpha \right]}_{D}^{24}$ − 10.93 (*c* 0.15, CH_3_OH, Table S1). An isomeric flavone derivative with a similar structure to **1** was reported from flowers of *Trollius chinensis* (Ranunculaceae), however, with the HMG position located at C-6 of a gly-2 galactosyl unit [33]. On the basic of all these data, **1** was identified as the yet undescribed 4″-(3-hydroxy-3-methylglutaroyl)-2″-*β*-D-glucopyranosyl vitexin.

Compound **2** was obtained as a brownish liquid from the ethyl acetate fraction. The molecular formula was deduced to be C_26_H_32_O_10_ based on the molecular ion peak at *m/z* 505.2036 ([M + H]^+^, calcd. 505.2068) in the positive ion HR-ESI-MS. The NMR data of compound **2** (Table 2, Figures S9 and S10) show the presence of an 3-(4-methoxyphenyl) propanoic acid moiety [34]. The structure of this part is the same as for compound **7**. Furthermore, NMR spectroscopic data also reveal similarities to those of 4-allyl-2,5-dimethoxyphenol-1-*β*-D-glucopyranoside (**11) [35]**, indicating that both compounds possess a similar structural unit except for the position of the glycosyl moiety.

**Figure 3:**
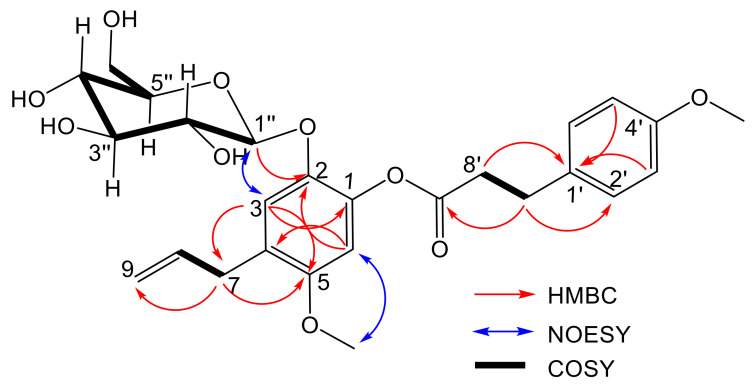
Key ^1^H ^1^H COSY, HMBC and NOESY correlation of compound **2**.

The HMBC long-range correlation (Figure 3) gave cross-peaks from the anomeric proton signal of the glycosyl moiety at δ = 4.58 (H-1″) to C-2 (δ_C_ 140.1), from the aromatic protons singlet at δ = 6.98 (H-3) to C-1 (δ_C_ 147.9) and C-5 (δ_C_ 155.3), from δ = 6.47 (H-6) to C-2 (δ_C_ 140.1), and C-4 (δ_C_ 120.7) and from the aliphatic proton at δ = 3.22 (H-7) to C-5 (δ_C_ 155.3) and C-3 (δ_C_ 121.8). This indicated that the glycosyl moiety is bound to C-2 and not at C-1 as found in **11.** This conclusion is supported by the NOESY correlation of H-1″ (δ_H_ 4.58) to H-3 (δ_H_ 6.98) and H-6 (δ_H_ 6.47) to 5-OMe (δ_H_ 3.73). Based on the HR-ESI-MS (Figure S16) and 1D- and 2D-NMR information (Figures S9–S14), compound **2** was determined as 4-allyl-5-methoxy-2-*β*-D-gluco- pyranosyloxy-3-(4-methoxyphenyl) propanoate, given the trival name kadukoside, based on the Malaysian species name kaduk. 

**Figure 4:**
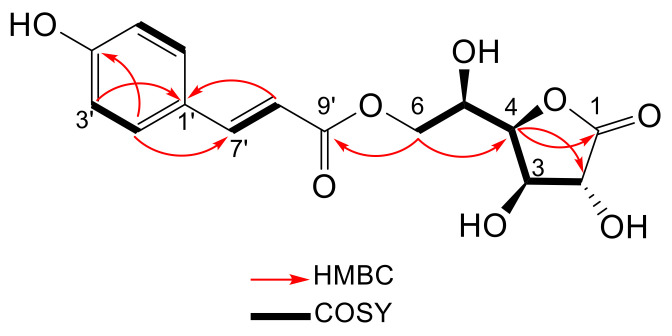
Key HMBC and ^1^H ^1^H COSY correlation of compound **3**.

Compound **3** was obtained as colorless liquid from the *n*-butanol fraction. The molecular formula was determined to be C_15_H_16_O_8_, based on HR-ESI-MS of the deprotonated ion at *m/z* 323.0769 (M − H)^−^, calcd. 323.0772 (Figure S25). The ^1^H NMR data showed the presence of *para*-substituted benzene ring proton signals [δ 7.47 (2H, d, *J* = 8.7 Hz, H-2′, 6′), 6.81 (2H, d, *J* = 8.7 Hz, H-3′, 5′)] and olefinic double bond proton signals [δ 7.67 (1H, d, *J* = 16 Hz, H-7′), [δ 6.38 (1H, d, *J* = 16 Hz, H-8′) (Table 3). In particular, the coupling constant value (*J* = 16 Hz) of the olefinic proton signals reveals a *trans* double bond. These NMR data supported the partial structure of **3** as a *trans-p-*coumaroyl group. An extensive analysis of 1D and 2D NMR experiments (Figures S17–S24) allowed the complete assignments of the protons and carbons of the sugar part. These assignments were further confirmed by COSY and 1D TOCSY correlation of sugar H-2 (δ_H_ 4.32), H-3 (δ_H_ 4.39), H-4 (δ_H_ 4.59), H-5 (δ_H_ 4.24) and H-6 (δ_H_ 4.35 and 4.44) (see Figures S22 and S23). The sugar moiety was identified as D-glucono-1,4-lactone by its coupling constants [36]. HMBC correlations observed from the protons at δ_H_ 4.35/4.44 (H-6A/6B) to C-9′ from the *trans-p-*coumaroyl group indicate the connectivity of both moieties. Based on these analyses, the structure of **3** was determined as shown in Figure 4. 

The relative stereochemistry of **3** was supported by NOESY correlation. This correlation of H-3 to H-4; H-4 to H-5; H-6B to H-5 suggested that H-3, H-4, H-5, and H-6B are positioned on the same side (see Figure S24). Furthermore, the compound showed positive optical rotation ${\left[\alpha \right]}_{D}^{23}$ + 4.04 (*c* 0.25, CH_3_OH) (Table S3). By comparison to the data reported by Tanaka and co-workers [37] (Table S2), who described a L-galactono-1,4-lactone derivative which was isolated from hops (*Humulus lupulus* L.), compound **3** is suggested to be 6-*O*-*trans-p-*coumaroyl-D-glucono-1,4-lactone.

In addition, ten of the known compounds, namely hydrocinnamic acid (**6**) [38], 3-(4-methoxyphenyl) propanoic acid (**7**) [34], methyl 3-(4-methoxyphenyl)propionate (**8**) [39], isoasarone (**12**) [40], *trans*-asarone (**15**) [40], cepharadione A (**21**) [41], piperolactam A (**23**) [42], loliolide (**28**) [43] and mix of stigmasterol (**33**) [44] and *β*-sitosterol (**34**) [44] were identified by comparison with literature data. These known compounds have already been isolated from *P. sarmentosum* [15,45,46]. 

Furthermore, twelve compounds, 5-hydroxy-7,4′-dimethoxyflavone (**4**) [47], benzyl-*β*-D-glucopyranoside (**9**) [48], 1-(2,4,5-trimethoxyphenyl)-1,2-propanedione (**13**) [49], 1′,2′-dihydroxyasarone (**14**) [50], asaraldehyde (**16**) [51], *trans*-*N*-feruloyltyramine (**18**) [52], paprazine (**19**) [52], norcepharadione B (**20**) [53], aristolactam B II (**22**) [54], reseoside (**29**) [55], andamanicin (**31**) [56], and magnosalin (**32**) [56] were previously isolated from other *Piper* species such as *P. capense* L.f. [57], *P. crocatum* Ruiz & Pav. [58], *P. cubeba* L. [59,60], *P. sumatranum* (Miq.) C. DC. [50], *P. puberulum* (Benth.) Maxim. [61], *P. ribesioides* Wall. [62], *P. clusii* (Miq.) C.DC. [50,63], and *P. aduncum* L. [64]. This indicated a close relationship among the *Piper* species. 

Moreover, another nine compounds are herein reported for the Piperaceae family for the first time. These compounds comprise the flavonoid-*C*-glycoside 2″-*O*-*β*-L-galactopyranosylvitexin (**5**) [65] that was previously isolated from *Trollius ledebouri* (Ranunculaceae). Three phenolic compounds, citrusin C (**10**) [66], 4-allyl-2,5-dimethoxyphenol-1-*β*-D-glucopyranoside (**11**) [35] and scoparone (**17**) [67] were reported from *Morina nepalensis* (Caprifoliaceae) [66], *Pelargonium sidoides* (Geraniaceae) [35] and *Jatropha multifidi* (Euphorbiaceae) [67], respectively. Furthermore, the four alkaloids dehydroformouregine (**24**) [68], 3-[2,3-dihydroxy-4-(hydroxymethyl)tetrahydrofuran-1-yl]-pyridine-4,5-diol (**25**) [69], naphthisoxazol A (**26**) [70] and indole-3-carboxaldehyde (**27**) [71] were previously isolated from *Guatteria ouregou* (Annonaceae) [68], *Tenebrio molitor* [69], *Glehnia littoralis* (Umbelliferae) [70], and *Isatis ingigotica* (Brassicaceae) [71], respectively. Lastly, a lignan, magnosalicin (**30**) [72] was found in *Magnolia* species (Magnoliaceae) [73]. The structures of these known compounds were identified by spectroscopic analyses and by comparison with data reported in the literature.

### 2.2. Biological Assays of Isolated Compounds

Earlier review studies of *P. sarmentosum* have reported diverse pharmacological activities, either as an extract or for some pure compounds [15]. Therefore, the compounds isolated from this species were tested for their anthelmintic, antifungal, antibacterial and cytotoxic properties. These biological examinations were conducted by using established model organisms that are non-pathogenic to humans and selected human cancer cell lines (Biosafety level-1) suitable for rapid screening assays. 

**Figure 5:**
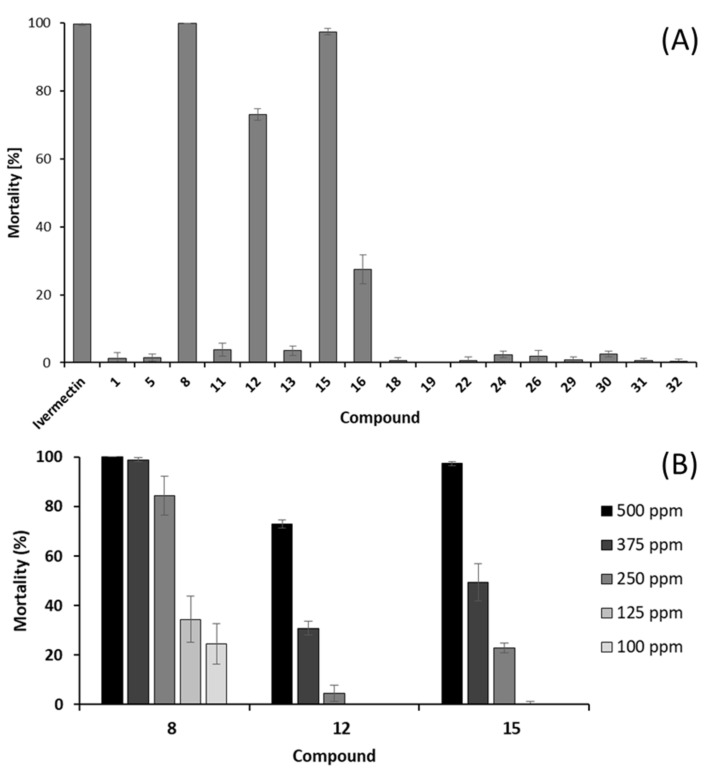
(**A**) Screening of anthelmintic activity against *Caenorhabditis elegans* of isolated compounds at 500 ppm. (**B**) Anthelmintic activity against *C. elegans* of methyl 3-(4-methoxyphenyl)propionate (**8**), isoasarone (**12**), and *trans*-asarone (**15**). Positive control ivermectin 10 μg/mL killed 100% of the nematodes. Mortality % based on three replicates.

The anthelmintic activity was evaluated against *Caenorhabditis elegans*. This biological screening demonstrated that three phenylpropanoids, methyl 3-(4-methoxyphenyl)-propionate (**8**), isoasarone (**12**), and *trans*-asarone (**15**) (^1^H NMR spectra in Figures S27–S29), show anthelmintic activity against *C. elegans* with 100.0 ± 0.0%, 73.0 ± 1.7%, and 97.4 ± 0.9% percentage mortality, respectively, at a test concentration of 500 ppm (Figure 5A). These promising compounds **8, 12**, and **15** were re-tested against *C. elegans* with different concentrations ranging from 500 ppm to 100 ppm in order to determine the concentration that kills 50% (LC_50_) of the nematodes. As shown in Figure 5B, the LC_50_ values were calculated (by in-house macro program in Microsoft Excel 2013) to be 174.6 ppm corresponding to 0.9 mM, 425.4 ppm/2.0 mM, and 341.9 ppm/1.6 mM, for compounds **8, 12**, and **15**, respectively. All three compounds are very unpolar constituents without free hydroxyl functions and accordingly were obtained from the *n*-hexane fraction. High lipophilicity is a prerequisite for transtegumental diffusion of anthelmintics [74]. 

The activity of *trans*-asarone (**15**) is in accordance with data published by McGaw et al. [75] on the anthelmintic activity against *C. elegans* of its isomer *β*-asarone isolated from *Acorus* species (Acoraceae). To the best of our knowledge, there are no reports on the anthelmintic activity of isoasarone (**12**) and methyl 3-(4-methoxyphenyl)propionate (**8**). However, isoasarone (**12**) has been reported to be toxic against the mosquitos *Aedes aegypti, Aedes albopictus* and *Culex quinquefasciatus* [40], while compound **8** exhibited strong antifeedant activity [76]. The obtained results provides scientific evidence of the anthelmintic activity of *P. sarmentosum* leaves, which are traditionally used to treat worm infections [16]. 

An earlier study indicated that *P. sarmentosum* extracts have antifungal properties [18]. However, there is lack of information on compounds responsible for that activity. Therefore, all isolated compounds were tested for their antifungal effects against the phytopathogenic ascomycetes *Botrytis cinerea* Pars, and *Septoria triciti* Desm. and the oomycete *Phytophthora infestans* (Mont.). Briefly, the isolated compounds were tested at a highest concentration of 125 μM, while the commercially available fungicides epoxiconazole and terbinafine at the same concentration as tested samples served as positive controls (Table 4). As shown in Table 4, seven compounds including the four phenylpropanoids derivatives kadukoside (**2**), methyl 3-(4-methoxyphenyl)propionate (**8**), isoasarone (**12**) and *trans*-asarone (**15**), the two alkaloids piperolactam A (**23**) and dehydroformouregine (**24**), and the flavonoid 5-hydroxy-7,4′-dimethoxyflavone (**4**) exhibited activity against the fungi *B. cinerea*, *S. tritici* or *P. infestans* with inhibition rates of about 40% at a concentration of 125 µM after seven days after inoculation. From these compounds, **2**, **12** and **15** possess an asarone skeleton.

**Figure 6:**
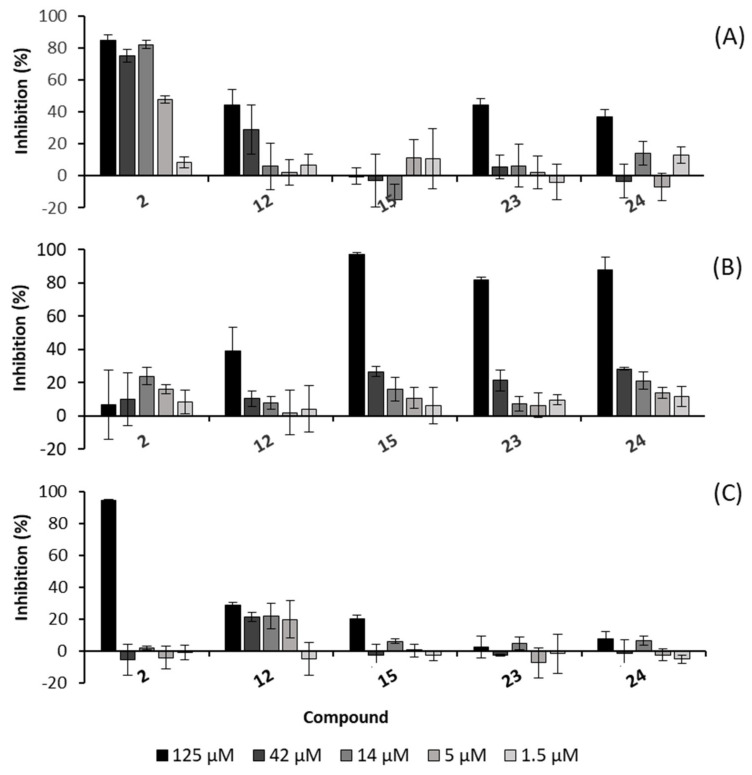
Antifungal activity of compounds **2**, **12**, **15**, **23**, and **24** against the phytopathogenic ascomycetes (**A**) *Septoria triciti*,(**B**) *Botrytis cinerea* and (**C**) *Phytophthora infestans*. Epoxiconazole in five different concentrations was used as a positive control for *S. triciti* and *B. cinerea* causing 100% inhibition, whereas terbinafine (125 µM) served as positive control for *P. infestans* inducing 100% inhibition after the inoculation period.

Based on the first hits determined at 125 µM concentration in the initial rapid-screening and sample availability, five compounds were subjected to dose-dependency studies against the fungi *S. tritici, B. cinerea* and *P. infestans*. The pathogens were treated with compound concentrations ranging from 1.5 to 125 µM, followed by assay read-out and data analyses. As displayed in Figure 6A, compound **2** was the most active one against *S. tritici* with inhibition rates of more than 75% at the concentrations of 14 µM and higher, and 48% inhibition at 5 µM. Thus, the IC_50_ of **2** calculated by SigmaPlot software was 5.0 ± 0.02 µM. Kadukoside (**2**) also displayed the highest inhibition activity against the oomycete *P. infestans* when treated at a concentration of 125 µM (Figure 6C). As depicted in Figure 6B, the remaining three compounds (**15, 23**, and **24**) exhibited significant activity against *B. cinerea*. with a dose-dependent effect from 1.5 to 125 µM. 

The results of compounds **23** and **24** suggest that the alkaloid scaffold is responsible for the antifungal activity. Similar antifungal activity was reported for structurally related piperolactam D and stigmalactam isolated from the aerial parts of *Piper parviflorum* C. DC. [77]. The substituents and their positions were suggested to be relevant for the distinct antifungal bioactivity [78]. Moreover, isoasarone (**12**) and *trans*-asarone (**15**) have been previously found in *P. sarmentosun* and other antifungal species such as *Boesenbergia pulcherrima* (Zingiberaceae) [79] and *Acorus species (Acoraceae)* [80]. Both of these compounds have been reported to possess antifungal activity against *Candida albicans* [81]. Surprisingly, in an earlier study *trans*-asarone did not show in vivo growth inhibition against *B. cinerea* at a concentration of 125 and even 1000 ppm [82]. 

**Figure 7:**
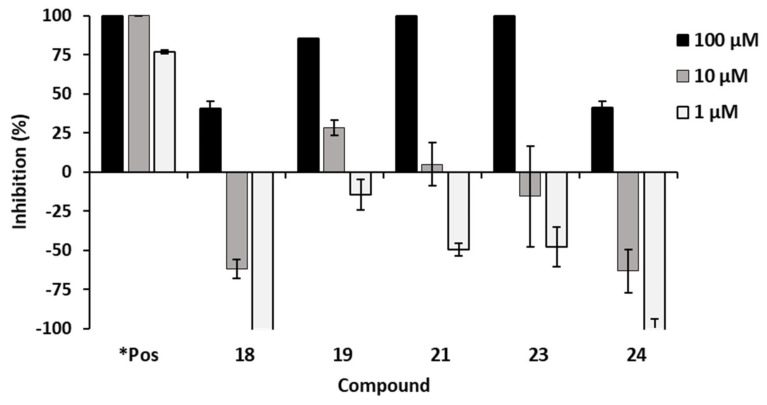
Antibacterial assay with compounds **18**, **19**, **21**, **23** and **24** against Gram-negative *Aliivibrio fischeri*. Chloramphenicol was used as the positive control labelled as *Pos. Negative values indicate an increase of bacterial growth in comparison to the negative control (0% inhibition).

Furthermore, all compounds **1**–**34** were screened for their antibacterial activity against the Gram-negative bacterium *Aliivibrio fischeri* at 1, 10 and 100 µM (Table 4). As indicated in Figure 7, five alkaloid compounds namely, *trans*-*N*-feruloyltyramine (**18**), paprazine (**19**), cepharadione A (**21**), piperolactam A (**23**) and dehydroformouregine (**24**), induced over 40% inhibition of bacterial growth at the highest concentration of 100 µM. Lower concentrations showed no inhibition or even promoted the bacterial growth. In general, alkaloids are nitrogen-containing organic compounds with often significant biological activities. They exist widely in the plant world [5].

The antibacterial activity of compound **19** with 85% inhibition at a concentration of 100 µM was about two-fold better than that of the structurally related compound **18** (41% inhibition). The observed differences in inhibition between the two compounds may be due to the presence of different functional groups at C-3 of the *p*-coumaroyl moiety. Our results of these two compounds were similar to those reported by Mata et al., which indicate that an extra methoxy group in the *p*-coumaroyl unit lowers the antibacterial activity [83]. Furthermore, compound **19** which was isolated previously from *Cannabis sativa* (Cannabaceae) roots has been reported to possess antibacterial activity against a different Gram-negative bacterium, *Escherichia coli* with an IC_50_ value of 0.8 µg/mL [84].

Moreover, the aporphines cepharadione A (**21**) and piperolactam A (**23**) almost completely inhibited the Gram-negative bacterium *A. fischeri* at the highest concentration of 100 µM. Contrary to our results, compound **21** which was isolated recently from the aerial parts of *Piper wallichii* (Miq.) Hand.-Mazz. showed a different trend in antibacterial activity. This compound was only active against the Gram-positive bacteria *Bacillus cereus*, *Bacillus subtilis* and *Staphylococcus aureus*; however, it was inactive against the three tested pathogenic Gram-negative bacteria *E. coli*, *Pseudomonas aeruginosa* and *Shigella sonnei* [85]. Thus, the compound may exhibit selective antibacterial activity against different Gram-positive and Gram-negative species. With regard to compound **23**, similar antibacterial properties have been described. For example, in an antimycobacterial bioassay-guided chromatographic study on *Piper sanctum* (Miq.) Schl. leaves performed by Mata et al. [83], compound **23** displayed good growth inhibition against *Mycobacterium tuberculosis* with an MIC value of 8 µg/mL. Compound **24** (dehydroformouregine) showed moderate antibacterial activity with 41% inhibition against the Gram-negative bacterium *A. fischeri* at 100 µM. Despite this compound having been previously isolated from *Guatteria ouregou* [68], there is no pharmacological activity reported, specifically no antibacterial activity; thus to the best of our knowledge, this is the first report of the antibacterial activity of compound **24**.

The cytotoxicity and impact of most isolated compounds on the metabolic cell viability at a reasonable concentration of 10 nM and 10 μM were exemplarily evaluated using HT-29 (human colorectal adenocarcinoma) and PC-3 (human prostate adenocarcinoma) cancer cell lines. The effect on the cancer cell viability was determined by conducting an MTT (3-(4,5-dimethylthiazol-2-yl)-2,5-diphenyltetrazolium bromide) assay. The reduction of the tetrazolium dye is assumed to depend on NAD(P)H-dependent mitochondrial oxidoreductases and reflects the metabolic activity of the cells. A high metabolic activity is connected to high proliferation. General cytotoxic effects were determined by using a Crystal Violet (CV) assay. CV is applied to stain adherent intact cells and thus indirectly indicates cell death. Both complementary assays were performed after 48 h treatment with the compounds under investigation. A very potent permeabilizer of cell membranes, digitonin (125 µM), was used as a positive control compromising the cells to the point of 0% of cell viability after 48 h. 

As demonstrated in Figure S30 of the supplementary data, most of the compounds tested did not reduce the cell viability below 80% even at the highest concentration of 10 µM. This indicates that compounds are very weakly or inactive against the specific cell lines. Meanwhile, compounds with cell viability below 80% at a concentration of 10 µM could be considered potentially cytotoxic and need further investigation. Briefly, five compounds including two alkaloids, aristolactam BII (**22**) and dehydroformouregine (**24**), and three neolignans, magnosalicin (**30**), andamanicin (**31**), and magnosalin (**32**), met this criterion for the HT-29 cell line either in MTT or CV assay at 10 µM. The observed cell viabilities in the MTT assay were 68.4 ± 1.5%, 76.4 ± 3.5% and 62.5 ± 4.2% for compounds **22**, **31** and **32**, respectively. While in the CV assay the cell viability was 52.9 ± 2.2%, 80.9 ± 5.9%, 71.7 ± 3.3%, 67.8 ± 2.2% and 61.7 ± 3.4% for compounds **22**, **24**, **30**, **31** and **32**, respectively. Interestingly, there were no significant variances in cytotoxicity between compounds **31** and **32,** which possess stereochemical differences in the position of methyl groups at C-1 and C-2. In addition, three alkaloid compounds, cepharadione A (**21**), aristolactam BII (**22**), and piperolactam A (**23**) reduced cell viability of the PC-3 cell line below 80% at the highest concentration of 10 µM. The IC_50_ values of all compounds can be estimated to be above 10 µM.

Out of these seven isolated compounds, only compounds **21**, **22** and **23** were previously tested against HT-29 cell lines. Compound **22** previously demonstrated cytotoxic effects with an IC_50_ value of 26 µg/mL [86], whereas for compounds **21** and **23** no activity was reported against HT-29 cell lines [87]. Furthermore, no data on **21**, **22** and **23** against the human prostate (PC-3) cancer cell line has been published.

Although cytotoxicity against HT-29 and PC-3 cell lines has not been reported for the neolignans **30**–**32**, magnosalicin (**30**) and magnosalin (**32**) have been tested against four human tumor cell lines, namely AS49 (non-small cell lung carcinoma), SK-OV-3 (ovary malignant ascites), SK-MEL-2 (skin melanoma), and HCT-15 (colon adenocarcinoma), showing IC_50_ above 8.5 µM [88]. However, these three compounds are known for neuroprotective and anti-inflammatory effects. Magnosalicin (**30**) was recently reported to exhibit significant anti-Aβ_42_ aggregation activity with an inhibitory rate of 61% at 100 µM, in contrast to 69% for the positive control EGCG [89]. Compounds **31** and **32** were previously isolated from the leaves of *Perilla frutescens* (Labiatae) and displayed inhibition of nitric oxide syntheses (IC_50_ 53.5 µM and 5.9 µM, respectively) and tumor necrosis factor-α in lipopolysaccharide-activated RAW 264.7 cells [56]. 

In summary, it is remarkable to note that the cell toxicity of the constituents is absent or low. This supports a safe usage of the species *P. sarmentosum* as food and medicinal plant. In accordance with the traditional application of this species, some isolated compounds from *P. sarmentosum* possess mild anthelmintic, antifungal, antibacterial or cytotoxic activities. It should be noted that impurities present in the isolated compounds may contribute to the observed effects. Nevertheless, the knowledge gained in this study on the molecular basis of *P. sarmentosum* will enable the future development of specific extracts and applications not only for human health but also for potential use in agriculture. For those constituents with a stronger effect (IC_50_ < 10 µM), a detailed mode of action for the specific biological activity should be addressed in future investigations.

## 3. Materials and Methods

### 3.1. General Methods

The following instruments were used for obtaining physical and spectroscopic data: Column chromatography was performed on silica gel (400–630 mesh, Merck, Germany), Sephadex LH-20 (Fluka, Steinheim, Germany) and Diaion HP20 (Supelco, Bellefonte, PA, USA). Fractions and substances were monitored by TLC. TLC was conducted on precoated Kieselgel 60 F 254 plates (Merck, Darmstadt, Germany) and the spots were detected either by examining the plates under an UV lamp at 254 and 366 nm or by treating the plates with vanillin or natural product reagents. The UV spectra were recorded on a Jasco V-770 UV-Vis/NIR spectrophotometer (Jasco, Pfungstadt, Germany), meanwhile specific rotation was measured with a Jasco P-2000 digital polarimeter (Jasco, Pfungstadt, Germany). 

NMR spectra were obtained with an Agilent DD2 400 system at +25 °C (Varian, Palo Alto, CA, USA) using a 5 mm inverse detection cryoprobe. The compounds were dissolved in CD_3_OD (99.8% D) or CDCl_3_ (99.8% D), and the spectra were recorded at 399.915 MHz (^1^H) and 100.569 MHz (^13^C). 1D (^1^H, ^13^C, and TOCSY) and 2D (^1^H,^13^C HSQC, ^1^H,^13^C HMBC, ^1^H-^1^H COSY, ^1^H,^1^H ROESY and ^1^H,^1^H NOESY) spectra were measured using standard CHEMPACK 8.1 pulse sequences implemented in the Varian VNMRJ 4.2 spectrometer software (Varian, Palo Al-to, CA, USA). ^1^H chemical shifts are referenced to internal TMS (^1^H δ = 0 ppm), while ^13^C chemical shifts are referenced to CD_3_OD (^13^C δ = 49.0 ppm) or CDCl_3_ (^13^C δ = 77.0 ppm). 

The semi-preparative HPLC was performed on a Shimadzu prominence system (Kyoto, Japan) equipped with LabSolutions software, LC-20AT pump, SPD-M20A diode array detector, SIL-20A auto sampler and FRC-10A fraction collector unit. Chromatographic separation was carried out using a YMC Pack C18 column (5 µm, 120 Å, 150 mm x 10 mm I.D, YMC, Devens, MA, USA) using H_2_O (A) and MeCN (B) as eluents at a flow rate of 2.2 mL/min. 

The high-resolution mass spectra in both positive and negative ion modes were acquired using either an Orbitrap Elite Mass spectrometer or API 3200 Triple Quadrupole System. The Orbitrap Elite Mass spectrometer (Thermofisher Scientific, Bremen, Germany) was equipped with an HRESI electrospray ion source (spray voltage 4.0 kV, capillary temperature 275 °C, source heater temperature 80 °C, FTMS resolution 100.000), whereas API 3200 Triple Quadrupole System (Sciex, Framingham, MA, USA) was equipped with a turbo ion spray source, which performs ionization with an ion spray voltage on 70 eV. During the measurement, the mass/charge range from 50 to 2000 was scanned.

### 3.2. Plant Material

The dried powdered leaves of *Piper sarmentosum* Roxb. were supplied by the Institute of Bioproduct Development, Universiti Teknologi Malaysia. The plant material was collected in January 2019 from Negeri Sembilan, Malaysia. A voucher was authenticated (Number: MFI 0039/19) by Dr. Mohd Firdaus Ismail, a botanist at the Institute of Biosciences, Universiti Putra Malaysia. A duplicate of the Herbarium specimen is kept at the Bioorganic Chemistry Department of the Leibniz Institute of Plant Biochemistry, Germany.

### 3.3. Extraction and Isolation

The dried leaf powder (400 g) was extracted five times (1.2 L/each) with 80% aqueous MeOH at room temperature. The combined extracts were concentrated under reduced pressure to obtain a crude MeOH extract (107 g). The extract was suspended in distilled H_2_O (250 mL) and partitioned with *n*-hexane, EtOAc and *n*-BuOH, yielding 18, 9 and 17 g of residue, respectively. Further fractionation of the remained aqueous fraction was not extended due to unpromising biological activity. The EtOAc-soluble fraction (9 g) was subjected to a silica gel column (Length, L = 40 cm; diameter, d = 5.5 cm) and eluted with a stepwise gradient of DCM:EtOAc:MeOH (1:0:0 to 0:0:1) to yield ten fractions (E1–E10). Fraction E3 (436 mg) underwent column chromatography (CC) over Sephadex LH-20 (L = 77 cm; d = 2.7 cm) eluted with MeOH to afford eight subfractions (E3a–E3h). Compound **27** (2.8 mg, *R*_f_ = 0.49, MeOH:DCM/1:9) was obtained from fraction E3e (2.8 mg). Fraction E3c (32.2 mg) was further purified by silica gel CC (L = 35 cm; d = 1.2 cm) eluting with *n*-hexane, DCM and MeOH in gradient elution to give compound **28** (2.3 mg, *R*_f_ = 0.51, MeOH:DCM/1:9). Compound **22** (1.9 mg, *R*_f_ = 0.67, MeOH:DCM/1:9) was isolated from fraction E3d (128.3 mg) via repeated silica gel CC (L = 60 cm; d = 1.0 cm) using a step gradient of *n*-hexane-DCM-MeOH (1:0:0 to 0:0:1). 

Fraction E5 (164 mg) was separated by a Sephadex LH-20 column (L = 72 cm; d = 1.5 cm) eluted with MeOH to produce seven subfractions (E5a–E5g). Compound **14** (17.5 mg, *R*_f_ = 0.54, MeOH:DCM/1:9) was afforded from fraction E5b (100.5 mg) over repeated silica CC (L = 35 cm; d = 1.2 cm) eluted with *n*-hexane:DCM:MeOH (2:8:0 to 0:9:1). Compound **23** (3.0 mg, *R*_f_ = 0.62, MeOH:DCM/1:9) was obtained from fraction E5f (3.0 mg). Further purification of fraction E5c (34.1 mg) using a silica gel column (L = 47 cm; d = 2.0 cm) in step gradient of *n*-hexane:DCM:MeOH (3:7:0 to 0:9:1) afforded compounds **18** (7.9 mg, *R*_f_ = 0.48, MeOH:DCM/1:9) and **19** (16.3 mg, *R*_f_ = 0.38, MeOH:DCM/1:9).

Fraction E6 (701 mg) was fractionated over a Sephadex LH-20 column (L = 77 cm; d = 2.7 cm) eluted with DCM/MeOH (1:1) yielding six subfractions (E6a–E6f). Fraction E6c (128 mg) was further purified using silica CC (L = 90 cm; d = 1.3 cm) in gradient elution of *n*-hexane:DCM:MeOH (1:0:0 to 0:7:3) to give 17 subfractions (E6c1–E6c17) including **21** (2.2 mg, *R*_f_ = 0.8, MeOH:DCM/1:9) and **7** (0.8 mg, *R*_f_ = 0.38, MeOH:DCM/1:9). Purification of fraction E6c15 (16.8 mg) on semipreparative RP-HPLC in a gradient system [H_2_O (A), MeCN (B); 0 min–10% B > 0–14 min 52% B > 14–16 min 52–100% B, flow rate 3.0 mL/min] afforded compounds **10** (1.9 mg, t_R_ = 10.7 min) and **11** (6.4 mg, t_R_ = 14.4 min). Compound **20** (0.6 mg, *R*_f_ = 0.66, MeOH:DCM/1:9) was obtained from fraction E6c8 (2.7 mg) by SPE cartridges (Chromabond@ C-18, 1ml/100 mg) eluted in a step gradient of H_2_O: MeOH (1:0 to 0:1). Fraction E6d (205 mg) and E6e (32 mg) were combined together and further fractionated via silica gel (L = 47 cm; d = 2.0 cm) in a step gradient of *n*-hexane:DCM:MeOH (3:7:0 to 0:9:1) affording nine subfractions (E6de1–E6de9). Compound **6** (2.4 mg *R*_f_ = 0.64, MeOH:DCM/1:9) was obtained from fraction E6de1 (4.9 mg) through SPE cartridges (Chromabond@ C-18, 1ml/100 mg) eluted with MeOH:H_2_O (1:9). Similarly, compound **2** (4.8 mg, *R*_f_ = 0.12, MeOH:DCM/1:9) was purified from E6de8 (19.2 mg) over a SPE cartridge (Chromabond@ C-18, 1ml/100 mg) eluted with MeOH:H_2_O (1:9). 

The *n*-hexane-soluble fraction (17 g) was subjected to silica gel CC (L = 40 cm; d = 5.5 cm) and eluted with a stepwise gradient of *n*-hexane:DCM:MeOH (1:0:0 to 0:7:3) to yield ten fractions (H1-H10). Compounds **8** (33.7 mg, *R*_f_ = 0.53, EtOAc:*n*-hexane/2.5:7.5), **12** (1453 mg, *R*_f_ = 0.49, EtOAc:*n*-hexane/2.5:7.5), **15** (1543 mg, *R*_f_ = 0.46, EtOAc:*n*-hexane/2.5:7.5), **16** (34.4 mg, *R*_f_ = 0.1, *n*-hexane:DCM/1:9) and **30** (7.0 mg, *R*_f_ = 0.17, EtOAc:*n*-hexane/3:7) were obtained from fraction H3 (4843 mg) via repeated column chromatographic separation using Sephadex LH-20 (L = 75 cm; d = 2.0 cm) eluted with DCM/MeOH (1:1) followed by silica gel (L = 75 cm; d = 2.0 cm) in a step gradient of *n*-hexane: EtOAc (9:1 to 4:6). Further separation of fraction H4 (965 mg) over Sephadex LH-20 (L = 40 cm; d = 5.5 cm) (DCM/MeOH, 1:1) afforded two subfractions (H4a–H4b). Mixture of compounds **33** and **34** (187 mg, *R*_f_ = 0.4, MeOH:DCM/5:9.5) were produced from fraction H4b by gradient elution of a silica column (L = 76 cm; d = 2.0 cm) using *n*-hexane:DCM:MeOH (1:0:0 to 0:9:1). Fraction H5 (704 mg) was subjected to Sephadex LH-20 CC (L = 76 cm; d = 3.3 cm) eluted with DCM/MeOH (1:1) to give four subfractions (H5a–H5d). Compounds **31** (46.2 mg, *R*_f_ = 0.16, EtOAc:*n*-hexane/3:7) and **32** (31.0 mg, *R*_f_ = 0.16, EtOAc:*n*-hexane/3:7) were afforded from fraction H5b (298 mg) via silica gel (L = 44 cm; d = 1.5 cm) eluted with *n*-hexane and EtOAc in a stepwise gradient (8:2 to 3:7). Fraction H5c (132 mg) was further purified over silica gel (L = 44 cm; d = 1.5 cm) eluted with *n*-hexane and EtOAc (8:2 to 0:1) to provide four subfractions (H5c1–H5c4). Compounds **17** (0.80 mg, t_R_ = 7.0 min) and **13** (0.85 mg, t_R_ = 8.8 min) were isolated from fraction H5c1 (5.4 mg) by semipreparative RP-HPLC using H_2_O (A) and MeCN (B) as eluent, and the gradient program was 0–12.5 min (25–50%B), 12.5–20 min (50–100%B), and 20–22 min (100%B). Further chromatography of fraction H5c3 (24.7 mg) via silica CC (L = 32 cm; d = 1.0 cm) eluted with *n*-hexane and EtOAc (1:1) yielded compound **24** (5.4 mg, *R*_f_ = 2.8, EtOAc:*n*-hexane/3:7). Compound **4** (13.2 mg, *R*_f_ = 0.52, EtOAc: *n*-hexane/1:1) was afforded from fraction H5c4 (13.2 mg).

The *n*-BuOH-soluble fraction was subjected to a Diaion-HP20 column (L = 60 cm; d = 2.8 cm) and eluted with an increasing solvent ratio of H_2_O and MeOH to give five fractions (B1–B5). Fraction B2 (1490 mg) was further purified over Sephadex LH-20 (L = 68 cm; d = 2.5 cm) eluted with MeOH to produce three subfractions (B2a–B2c). Compound **25** (13.3 mg, *R*_f_ = 0.34, MeOH:EtOAc/2:8) was yielded from fraction B2a (30.3 mg) by silica CC (L = 40 cm; d = 1.5 cm) (EtOAc: MeOH, 8:2 to 7.5:2.5). Fraction B2b (952 mg) was chromatographed via RP-18 CC (L = 55 cm; d = 2.1 cm) using H_2_O and MeOH as eluent, and the gradient elution was 10 to 50% of MeOH to afford six subfractions (B2b1–B2b6). Compound **26** (28.7 mg, *R*_f_ = 0.45, MeOH:EtOAc/7:3) was afforded from fraction B2b5 (411 mg) by silica gel CC (L = 43 cm; d = 2.5 cm) with EtOAc:MeOH (1:1 to 0.5:9.5). Separation of fraction B3 (4749 mg) via Sephadex LH-20 CC (L = 68 cm; d = 2.5 cm) eluted with MeOH gave four subfractions (B3a–B3d). Further purification of fraction B3a (1366 mg) using silica CC (L = 65 cm; d = 2.0 cm) eluted with a stepwise gradient of EtOAc:MeOH (9:1 to 4.5:5.5) produced 12 subfractions (B3a1–B3a12). Compound **29** (38.2 mg, *R*_f_ = 0.69, MeOH:EtOAc/3:7) was afforded from fraction B3a1 (38.2 mg). Fraction B3a9 (81 mg) was further separated over semipreparative RP-HPLC and eluted with a mixture of H_2_O (A) and MeCN (B), and the gradient elution was 0–12.5 min (10–48% B) and 12.5–13.5 min (48% B) to give sub-fraction 9a (13.6 mg, t_R_ = 6.2 min). Further purification was performed on sub-fraction 9a by semipreparative RP-HPLC with H_2_O (A) and 0.1% of TFA in MeOH (B) as mobile phase with the gradient program 0–23 min (22–50% B) and 23–24 min (50–100% B) to afford compound **1** (2.0 mg, t_R_ = 19.9 min). Fraction B3b was subjected to silica CC (L = 73 cm; d = 2.3 cm) eluted with EtOAc:MeOH (4:1 to 1:1) to obtain eight subfractions (B3b1–B3b8). Fraction B3b2 (95 mg) was purified via semipreparative RP-HPLC using H_2_O (A) and MeCN (B) as eluents. The gradient program was 0–15 min (10–29%B), 15–17 min (29–100%B) and 17–20 min (100%B) to yield eight subfractions B3b2a–B3b2h including compound **3** (10.3 mg, t_R_ = 12.8 min) from B3b2g (10.3 mg). Compound **9** (3.7 mg, t_R_ = 11 min) was purified from fraction B3b2a (16.0 mg) over modified RP-HPLC method via H_2_O and MeCN as eluent (92:8, isocratic elution, 3 mL/min). Compound **5** (226 mg, *R*_f_ = 0.26, MeOH:EtOAc/4:6) was isolated from fraction B3b5 (226 mg). 

Compound **1**: 4″-(3-Hydroxy-3-methylglutaroyl)-2″-*β*-D-glucopyranosyl vitexin. Brownish liquid; ${\left[\alpha \right]}_{D}^{24}$ -10.93 (*c* 0.15, CH_3_OH); UV λ_max_, (MeOH) (log e) 202 (5.0), 271 (5.1), 335 (5.2) (Figure S8); ^1^H-NMR (400 MHz, CD_3_OD): Table 1; ^13^C-NMR (100 MHz, CD_3_OD): Table 1; negative ion HR-ESI-MS [M − H]^−^ at *m/z* 737.1937, (calcd. for C_33_H_37_O_19_: 737.1935) (Figure S7). 

Compound **2**: 4-Allyl-3-methoxyphenol-6-*β*-D-glucopyranoside-3-(4-methoxyphenyl) propanoic acid (kadukoside). Brownish liquid; UV λ_max_, (MeOH) (log e) 202 (4.75), 223 (4.80), 384 (5.03) (Figure S15); ^1^H-NMR (400 MHz, CD_3_OD): Table 2; ^13^C-NMR (100 MHz, CD_3_OD): Table 2; positive ion HR-ESI-MS [M + H]^+^ peak at *m/z* 505.2036, (calcd. for C_26_H_32_O_10_: 505.2068) (Figure S16).

Compound **3**: 6-*O*-*trans-p-*Coumaroyl-D-glucono-1,4-lactone. Colorless liquid; ${\left[\alpha \right]}_{D}^{23}$ + 4.04 (*c* 0.25, CH_3_OH); UV λ_max_, (MeOH) (log e) 210 (4.4), 226 (4.5), 312 (4.6) (Figure S26); ^1^H-NMR (400 MHz, CD_3_OD): Table 3; ^13^C-NMR (100 MHz, CD_3_OD): Table 3; negative ion HR-ESI-MS [M-H]^-^ *m/z* 323.0769, (calcd. for C_15_H_16_O_8_: 323.0772) (Figure S25).

### 3.4. Biological Activities

#### 3.4.1. Anthelmintic Assay

The anthelmintic bioassay was conducted using the Bristol N2 wild type strain of the model organism *Caenorhabditis elegans*, which was previously demonstrated to correlate with anthelmintic activity against parasitic trematodes [90]. The nematodes were cultured on NGM (Nematode Growth Media) Petri plates using the uracil auxotroph *E. coli* strain OP50 as food source according to the methods described by Stiernagle [91]. The anthelmintic bioassay was performed according to method developed by Thomsen et al. [90]. The solvent DMSO (2%) and the standard anthelmintic drug ivermectin (10 μg/mL) were used as negative and positive controls in all the assays, respectively. All assays were performed in triplicate.

#### 3.4.2. Antifungal Assay

The antifungal activity was performed against phytopathogenic ascomycetes *Botrytis cinerea* Pars, and *Septoria triciti* Desm. and the oomycete *Phytophthora infestans* (Mont.) de Bary in 96-well microtiter plate assays with minor modification as described by Otto et al. [92]. In brief, the isolated compounds were tested at a highest concentration of 125 μM, while solvent DMSO was serving as negative control (max. concentration 2.5%), and the commercially available fungicides epoxiconazole and terbinafine (Sigma-Aldrich, Damstadt, Germany) served as reference compounds. The pathogen growth was assessed seven days after inoculation by the optical density (OD) at ʎ 405 nm measurement with a TecanGENios Pro microplate reader (five measurements per well using multiple reads in 3 × 3 square). Each experiment was performed in triplicates.

#### 3.4.3. Antibacterial Assay against *Aliivibrio fischeri*

The isolated compounds were tested at concentrations of 1 and 100 μM against the Gram-negative *Aliivibrio fischeri* test strain DSM507 (batch no. 1209), with chloramphenicol (100 μM) serving as a positive control as previously reported [3,93].

In brief, 25 mL BOSS medium containing fresh glycerol was incubated at 100 rpm and 23 °C for 16 to 18 h before being diluted with fresh BOSS medium to an appropriate cell number (luminescence value between 30,000 and 50,000 RLU). The assay was carried out in 96-well black flat-bottomed plates (Brand cell GradeTM premium, STERILE R) with a final volume of 200 μL of BOSS medium containing 1% DMSO per well (100 μL of diluted bacterial solution and 100 μL of test solution). The plates were incubated in the dark for 24 h without a lid and without shaking at a temperature of 23 °C and a humidity of 100 percent. The bioluminescence (measured in relative luminescence units, RLU) is proportional to cell density and was calculated after 24 h using the TecanGeniosPro microplate reader. As a result, the entire 1000 ms wavelength range was detected without any preliminary shaking to avoid secondary oxygen effects. The results (mean standard deviation value, n = 6) are given as relative values (percent inhibition) to the negative control (bacterial growth, 1 % DMSO without test compound). Negative values indicate that bacterial growth is accelerating or that luminescence is increasing.

#### 3.4.4. Cytotoxicity Assay

The cytotoxicity and impact on the metabolic cell viability of isolated compounds at 10 nM and 10 μM was evaluated against PC-3 (human prostate adenocarcinoma) and HT-29 (human colorectal adenocarcinoma) cancer cell lines. Both cell lines were purchased from ATCC (Manassas, VA, USA). The cell culture medium RPMI 1640, the supplements FCS and L-glutamine, as well as PBS and trypsin/EDTA were purchased from Capricorn Scientific GmbH (Ebsdorfergrund, Germany). Culture flasks, multi-well plates and further cell culture plastics were from Greiner Bio-One GmbH (Frickenhausen, Germany) and TPP (Trasadingen, Switzerland), respectively. PC-3 and HT-29 cells were cultured in RPMI 1640 medium supplemented with 10% heat-inactivated FCS, 2 mM L-glutamine and 1% penicillin/streptomycin, and in a humidified atmosphere with 5% CO_2_ at 37 °C. Routinely, cells were cultured in T-75 flasks until reaching subconfluency (~80%), subsequently cells were harvested by washing with PBS and detached by using trypsin/EDTA (0.05% in PBS) prior to cell passaging and seeding for sub-culturing and assays in 96-well plates [94].

The cell handling and assay techniques were in accordance to the method with minor modification as described by Khan et al. [94]. In brief, anti-proliferative and cytotoxic effects of the compounds were investigated by performing colorimetric MTT (3-(4,5-dimethylthiazol-2-yl)-2,5-diphenyltetrazolium bromide) and CV (crystal violet)-based cell viability assays (Sigma-Aldrich, Taufkirchen, Germany), respectively. For this purpose, cells were seeded in low densities in 96-well plates using the aforementioned cell culture medium. The cells were allowed to adhere for 24 h, followed by the 48 h compound treatment. Based on 20 mM DMSO stock solutions, the compounds were diluted in standard growth media to reach final concentrations of 10 nM and 10 μM for cell treatment. For control measures, cells were treated in parallel with 125 µM digitonin (positive control, for data normalization set to 0% cell viability). Each data point was determined in technical quadruplicates and two independent biological replicates. As soon as the 48 h incubation was finished, cell viability was measured.

For the MTT assay, cells were washed once with PBS, followed by incubation with MTT working solution (0.5 mg/mL MTT in culture medium) for 1 h under standard growth conditions. After discarding the MTT solution, DMSO was added in order to dissolve the formed formazan, followed by measuring formazan absorbance at 570 nm, and additionally at the reference/background wavelength of 670 nm, by using a SpectraMax M5 multi-well plate reader (Molecular Devices, San Jose, CA, USA).

For the CV assay, cells were washed once with PBS and fixed with 4% paraformaldehyde (PFA) for 20 min at room temperature (RT). After discarding the PFA solution, the cells were left to dry for 10 min and then stained with 1% crystal violet solution for 15 min at RT. The cells were washed with water and were dried overnight at RT. Afterwards, acetic acid (33% in ultrapure water) was added to the stained cells and absorbance was measured at 570 nm and 670 nm (reference wavelength) using a SpectraMax M5 multi-well plate reader (Molecular Devices, San Jose, CA, USA). For data analyses, GraphPad Prism version 8.0.2, SigmaPlot 14.0 and Microsoft Excel 2013 were used. The results are shown as a percentage of the control values obtained from untreated cultures, i.e., cell viability in percent.

## 4. Conclusions

This study represents the most comprehensive phytochemical characterization of *P. sarmentosum* which is used as medicinal and food plant in Asian countries. Investigation of *P. sarmentosum* leaves yielded three new compounds (**1**–**3**) and 31 known ones. Interestingly, 21 of these known compounds were isolated from this species for the first time and thus were not described in previous studies of *P. sarmentosum*. The structures of all compounds were confirmed by several spectroscopic techniques, i.e ^1^H-NMR, ^13^C-NMR, 2D NMR, and HRMS. For the first time all isolated compounds were evaluated for their anthelmintic, antifungal, antibacterial and cytotoxic activities to extend the knowledge of their biological effects. It is noteworthy that only very few compounds showed cytotoxic effects, and only at high concentration implying a safe consumption of this species. Some isolated compounds showed anthelmintic, antifungal and antibacterial potential in accordance with the traditional application of the plant species. Our finding suggests that *P. sarmentosum* can be used as an important source of mild health-promoting effects. 

## Figures and Tables

**Table 1:** ^1^H- and ^13^C-NMR data of **1** (in CD_3_OD; δ in ppm, *J* in Hz).

Pos.	δ_H_ ^a^, Mult. *J* (Hz)	δ_C_ ^b^	Pos.	δ_H_ ^a^, Mult. *J* (Hz)	δ_C_ ^b^
2		166.5	5″	3.66 m	80.7
3	6.62 s	103.6	6″A	3.77	62.7
4		184.2	6″B	3.67	62.7
5		162.7	1‴		172.2
6	6.24 s	99.4	2‴A	2.83	46.2
7		164.5	2‴B	2.73	46.2
8		104.9	3‴		70.9
9		158.2	4‴A	2.83	46.4
10		105.7	4‴B	2.72	46.4
1′		123.5	5‴		174.8
2′	8.04 d 8.7	130.1	3‴-CH_3_	1.46 s	27.7
3′	7.03 d 8.7	117.2	1″″	4.26 d 7.7	105.3
4′		162.9	2″″	2.97 t 8.0	75.7
5′	7.03 d 8.7	117.2	3″″	3.15 m	77.6
6′	8.04 d 8.7	130.1	4″″	3.11 m	71.3
1″	5.09 d 10.1	73.8	5″″	2.76 m	77.0
2″	4.42 t 9.4	80.7	6″″A	3.41	62.4
3″	3.96 m	77.7	6″″B	3.31	64.4
4″	5.26 t 9.6	72.7			

^a^ Recorded 400 MHz. ^b^ Recorded at 100 MHz.

**Table 2:** ^1^H- and ^13^C-NMR data of **2** (in CD_3_OD; δ in ppm, *J* in Hz).

Pos.	δH ^a^, Mult. *J* (Hz)	δ_C_ ^b^	Pos.	δH ^a^, Mult. *J* (Hz)	δ_C_ ^b^
1	-	147.9	3′/5′	6.81 d 8.6	114.9
2	-	140.1	4′	-	159.6
3	6.98 s	121.8	7′	2.84 t 7.7	31.3
4	-	120.7	8′	2.54 t 7.7	37.2
5	-	155.3	9′	-	176.9
6	6.47 s	100.9	4′-OMe′	3.75	55.2
7	3.22 d 6.4	34.6	1″	4.58 d 7.2	106.0
8	5.91 ddt 16.7, 9.7, 6.4	138.7	2″	3.44 m	75.0
9A	5.00 dd 16.7, 1.9	115.2	3″	3.42	77.7
9B	4.94 dd 9.7, 1.9	115.2	4″	3.30 m	71.2
5-OMe	3.73	56.2	5″	3.34	78.2
1′	-	134.2	6A″	3.71	62.3
2′/6′	7.12 d 8.6	130.3	6B″	3.87	62.3

^a^ Recorded at 400 MHz. ^b^ Recorded at 100 MHz.

**Table 3:** ^1^H- and ^13^C-NMR data of **3** (in CD_3_OD; δ in ppm, *J* in Hz).

Pos.	δH ^a^, Mult. *J* (Hz)	δ_C_ ^b^	Pos.	δH ^a^, Mult. *J* (Hz)	δ_C_ ^b^
1		177.1	2′	7.47 d 8.7	131.2
2	4.32 d 4.7	74.8	3′	6.81 d 8.7	116.8
3	4.39 dd 5.4, 4.7	74.9	4′		161.3
4	4.59 dd 6.3, 5.4	81.3	5′	6.81 d 8.7	116.8
5	4.24 ddd 6.3, 6.3, 3.3	69.3	6′	7.47 d 8.7	131.2
6A	4.35 dd 11.7, 3.3	66.9	7′	7.67 d 16.0	146.9
6B	4.44 dd 11.7, 6.3	66.9	8′	6.38 d 16.0	114.9
1′		127.2	9′		169.1

^a^ Recorded at 400 MHz. ^b^ Recorded at 100 MHz.

**Table 4:** Antifungal (*Botrytis cinerea, Septoria tritici*), antioomycotic (*Phytophthora infestans*), and antibacterial (*Aliivibrio fischeri*) activities of isolated compounds from *P. sarmentosum*.

	Antifungal Assays			Antibacterial Assays
	Growth Inhibition [%] ^a^			Growth Inhibition [%] ^a^
	*B. cinerea*	*S. tritici*	*P. infestans*	*A. fischeri*
**Compound**	125 µM	125 µM	125 µM	100 µM ^b^
**1**	−18.6 ± 13.4	14.6 ± 15.5	−10.7 ± 3.0	−13.9 ± 11.4
**2**	6.8 ± 20.5	84.9 ± 3.4	94.4 ± 0.7	−33.2 ± 19.3
**3**	15.6 ± 6.7	12.1 ± 9.9	4.2 ± 2.4	8.2 ± 12.1
**4**	7.7 ± 11.1	48.3 ± 4.2	−39.6 ± 8.5	2.1 ± 9.7
**5**	−49.9 ± 9.4	22.3 ± 6.6	−12.4 ± 9.5	−7.1 ± 13.6
**6**	20.8 ± 6.3	−33.9 ± 16.4	−9.2 ± 9.4	−41.4 ± 19.8
**7**	29.9 ± 10.3	−11.7 ± 14.5	1.1 ± 2.0	−34.67 ± 26.3
**8**	48.4 ± 10.9	44.9 ± 8.3	−31.0 ± 13.0	−3.3 ± 9.2
**9**	21.6 ± 5.1	−7.7 ± 8.1	−12.6 ± 9.3	−43.5 ± 12.9
**10**	1.9 ± 6.9	13.2 ± 7.3	3.1 ± 2.8	−4.7 ± 11.7
**11**	20.1 ± 4.7	42.3 ± 1.9	0.4 ± 10.0	−15.4 ± 10.3
**12**	39.2 ± 14.3	44.1 ± 1.9	28.5 ± 1.8	−73.9 ± 27.4
**15**	96.9 ± 1.6	−0.1 ± 5.2	20.0 ± 2.5	−49.4 ± 6.4
**16**	8.3 ± 6.1	6.7 ± 5.6	−5.4 ± 9.1	6.2 ± 7.6
**18**	5.2 ± 1.6	0.8 ± 6.8	−18.1 ± 17.9	40.7 ± 4.8
**19**	2.6 ± 6.2	−7.5 ± 6.9	−10.3 ± 5.5	85.4 ± 0.2
**21**	33.9 ± 9.0	6.3 ± 23.8	0.8 ± 1.6	100. ± 0.1
**22**	−36.3 ± 24.5	−7.1 ± 16.3	−31.5 ± 16.7	−53.3 ± 15.6
**23**	81.9 ± 1.7	44.5 ± 3.9	2.4 ± 6.8	99.6 ± 0.3
**24**	87.8 ± 7.5	36.8 ± 4.5	7.5 ± 4.3	41.1 ± 3.9
**25**	28.4 ± 5.5	−1.8 ± 7.5	27.2 ± 13.9	−16.2 ± 8.6
**26**	32.9 ± 12.5	−13.5 ± 10.7	−7.8 ± 6.6	−91.7 ± 16.1
**28**	30.4 ± 3.8	12.1 ± 17.4	3.1 ± 3.5	−55.5 ± 19.1
**29**	0.4 ± 4.8	34.5 ± 6.4	−35.7 ± 0.6	−5.3 ± 16.6
**30**	32.8 ± 5.8	−1.9 ± 13.7	−22.9 ± 4.6	14.1 ± 13.0
**31**	37.7 ± 3.1	−25.1 ± 20.2	−19.5 ± 10.7	8.4 ± 4.1
**32**	34.8 ± 6.6	−31.1 ± 29.3	−18.6 ± 7.1	−14.5 ± 6.8
Pos. control	125 µM epoxiconazole		125 µM terbinafine	100 µM chloramphenicol
	92.0 ± 1.4		96.8 ± 1.2	99.0 ± 0

^a^ Negative values indicate an increase of fungal or bacterial growth in comparison to the negative control (0% inhibition). ^b^ Growth inhibition rates below 50% indicate IC_50_ values > 100 µM.

## Data Availability

The data presented in this study are available on request from the corresponding author.

## Supplementary Materials

The following supporting information can be downloaded at: https://www.mdpi.com/article/10.3390/ijms24021328/s1.
