# Supplementary material for: Characterization and Bioactive Potential of Secondary Metabolites Isolated from Piper sarmentosum Roxb
Source: Int J Mol Sci. 2023 Jan 10;24(2):1328. doi: 10.3390/ijms24021328 (PMC9862425; doi:10.3390/ijms24021328)
Supplement: Supplementary file 1 [file ijms-24-01328-s001.zip › ijms-2101665-supplementary.pdf]

Supporting Information

Characterization and bioactive potential of secondary metabolites isolated from *Piper sarmentosum* Roxb.

Ismail Ware[1,2], Katrin Franke[1,3,4*], Mthandazo Dube[1], Hesham Ali El Enshasy[2,5] and Ludger A. Wessjohann[2,4*]

[1] Department of Bioorganic Chemistry, Leibniz Institute of Plant Biochemistry, D-06120 Halle (Saale), Germany
[2] Institute of Bioproduct Development, Universiti Teknologi Malaysia (UTM), 81310 Johor Bahru, Johor, Malaysia
[3] Institute of Biology/Geobotany and Botanical Garden, Martin Luther University Halle-Wittenberg, 06108 Halle (Saale), Germany
[4] German Centre for Integrative Biodiversity Research (iDiv) Halle-Jena-Leipzig, 04103 Leipzig, Germany
[5] City of Scientific Research and Technology Applications (SRTA), New Borg Al Arab, Alexandria 21934, Egypt

* Correspondence: kfranke@ipb-halle.de (K.F.); wessjohann@ipb-halle.de (L.A.W.) Tel.: +49-345-5582-1380 (K.F.); +49-345-5582-1300 (L.A.W.)

TABLE OF CONTENTS **PAGE**

Figure S1: ^{1}H NMR spectrum of compound **1** (400 MHz, MeOH-*d*4) ... 2
Figure S2: ^{13}C NMR spectrum of compound **1** (100 MHz, MeOH-*d*4) ... 2
Figure S3: HSQC spectrum of compound **1** (400 MHz, MeOH-*d*4) ... 3
Figure S4: HMBC spectrum of compound **1** (400 MHz, MeOH-*d*4) ... 3
Figure S5: ^{1}H-^{1}H COSY spectrum of compound **1**; A) full spectrum, B) at glycosides region (400 MHz, MeOH-*d*4) ... 4
Figure S6: 2D-ROESY spectrum at glucosides region of compound **1** (400 MHz, MeOH-*d*4) ... 4
Figure S7: HR-ESI-MS spectrum of compound **1** in negative ion mode ... 5
Figure S8: UV spectrum of compound **1** in MeOH ... 5
Table S1: Polarimeter data of compound **1**. ... 6
Figure S9: ^{1}H NMR spectrum of compound **2** (400 MHz, MeOH-*d*4) ... 7
Figure S10 : ^{13}C NMR spectrum of compound **2** (400 MHz, MeOH-*d*4) ... 7
Figure S11: HSQC spectrum of compound **2** (400 MHz, MeOH-*d*4) ... 8
Figure S12: HMBC spectrum of compound **2** (400 MHz, MeOH-*d*4) ... 8
Figure S13: 2D-NOESY spectrum of compound **2** (400 MHz, MeOH-*d*4) ... 9
Figure S14: ^{1}H-^{1}H COSY spectrum of compound **2** (400 MHz, MeOH-*d*4) ... 9
Figure S15: UV spectrum of compound **2** in MeOH ... 10
Figure S16: HR-ESI-MS spectrum of compound **2** in positive ion mode ... 10
Figure S17: ^{1}H NMR spectrum of compound **3** (400 MHz, MeOH-*d*4) ... 11
Figure S18: ^{13}C NMR spectrum of compound **3** (100 MHz, MeOH-*d*4) ... 11
Figure S19: DEPT135 spectrum of compound **3** (100 MHz, MeOH-*d*4) ... 12
Figure S20: HSQC spectrum of compound **3** (400 MHz, MeOH-*d*4) ... 12
Figure S21: HMBC spectrum of compound **3** (400 MHz, MeOH-*d*4) ... 13
Figure S22: ^{1}H-^{1}H COSY spectrum of compound **3** (400 MHz, MeOH-*d*4) ... 13
Figure S23: 1D-TOCSY spectrum of compound **3** with selective excitation of H-4 (δ_H 4.59) and mixing times (400 MHz, MeOH-*d*4) ... 14
Figure S24: 2D-NOESY spectrum of compound **3**; A) full spectrum, B) glycoside region (400 MHz MeOH-*d*4) ... 14
Table S2: ^{1}H and ^{13}C NMR comparison data of compound **3** and similar structure (CD_3OD). ... 15
Figure S25: HR-ESI-MS spectrum of compound **3** in negative ion mode ... 15
Figure S26: UV spectrum of compound **3** in MeOH ... 16
Table S3: Polarimeter data of compound **3**. ... 17
Figure S27: ^{1}H NMR spectrum of compound **8** (400 MHz, $CDCl_3$) ... 18
Figure S28: ^{1}H NMR spectrum of compound **12** (400 MHz, $CDCl_3$) ... 18
Figure S29: ^{1}H NMR spectrum of compound **15** (400 MHz, $CDCl_3$) ... 19
Figure S30: Antiproliferative and cytotoxic activities (MTT and CV) of compounds isolated from *P. sarmentosum* against human colorectal (HT-29) and prostate (PC-3) cancer cell lines. ... 20

4''-(3-Hydroxy-3-methylglutaroyl)-2''-*β*-D-glucopyranosyl vitexin

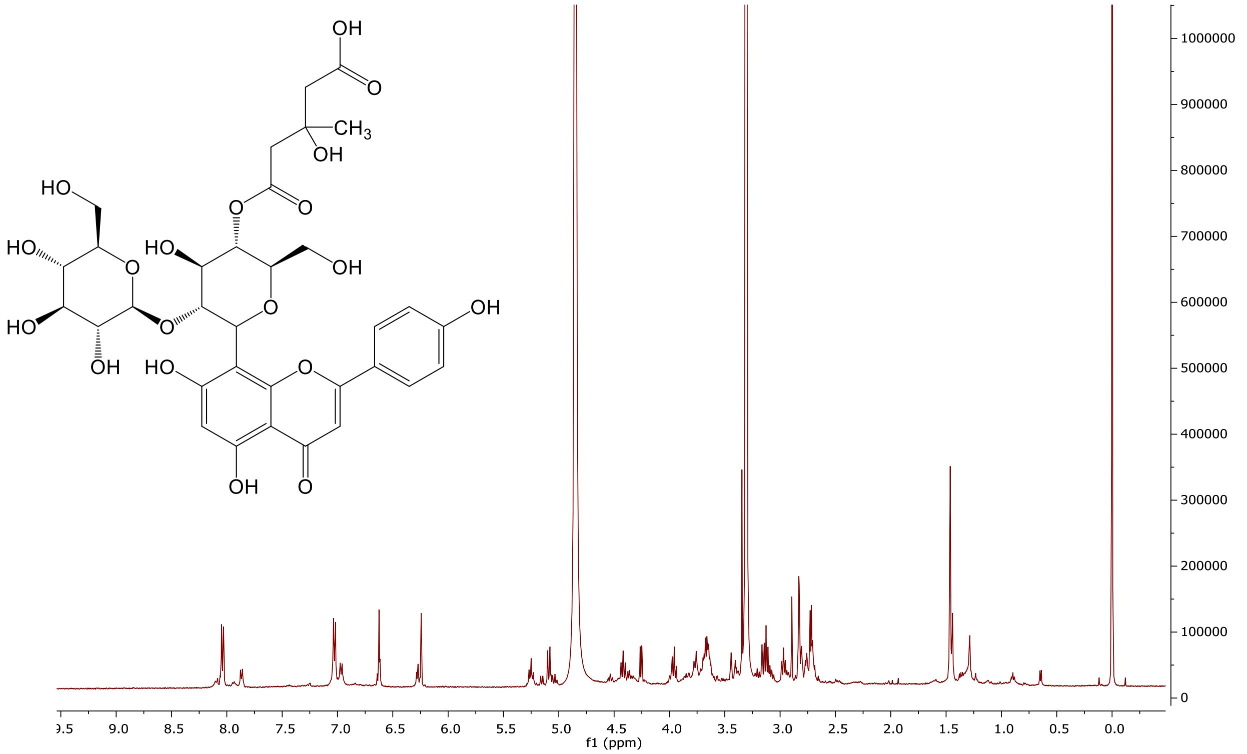

Figure S1: ^{1}H NMR spectrum of compound **1** (400 MHz, MeOH-*d*4)

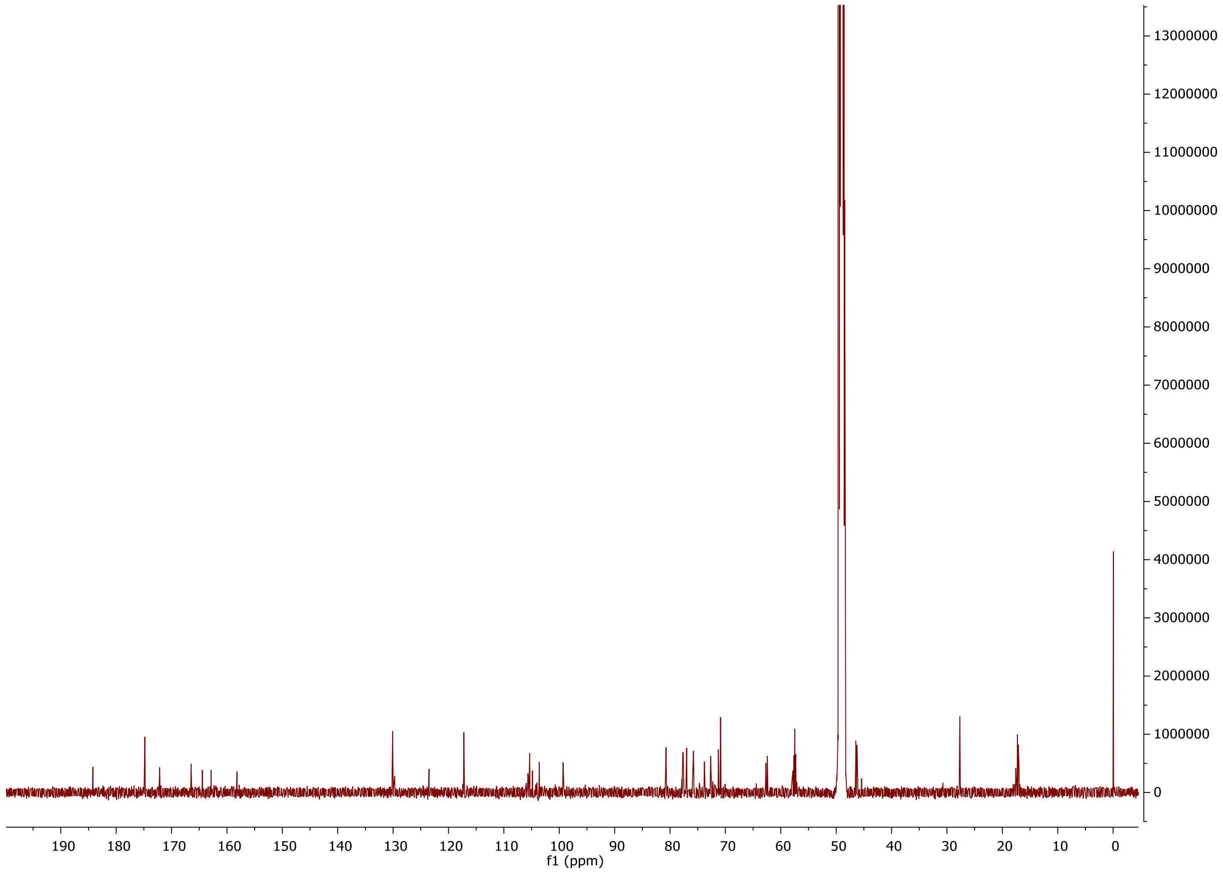

Figure S2: ^{13}C NMR spectrum of compound **1** (100 MHz, MeOH-*d*4)

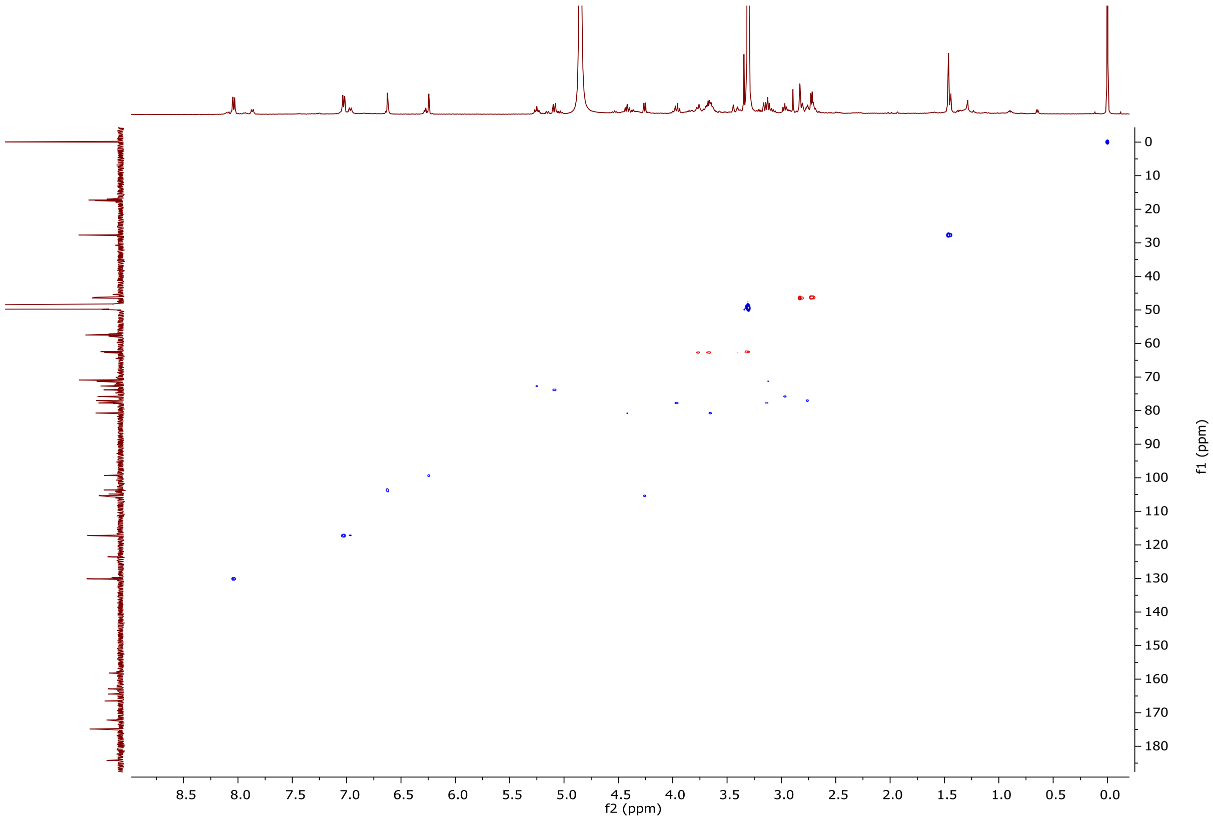

Figure S3: HSQC spectrum of compound **1** (400 MHz, MeOH-*d*4)

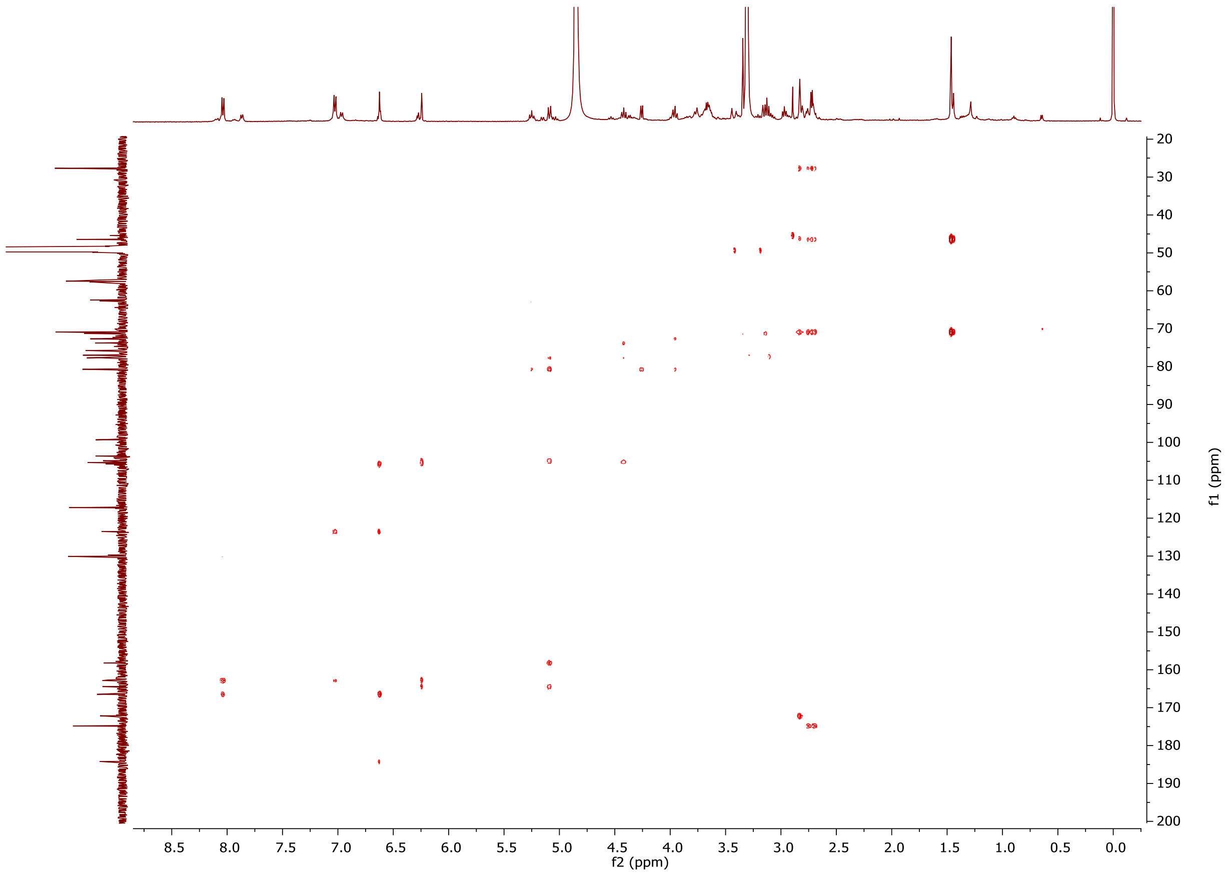

Figure S4: HMBC spectrum of compound **1** (400 MHz, MeOH-*d*4)

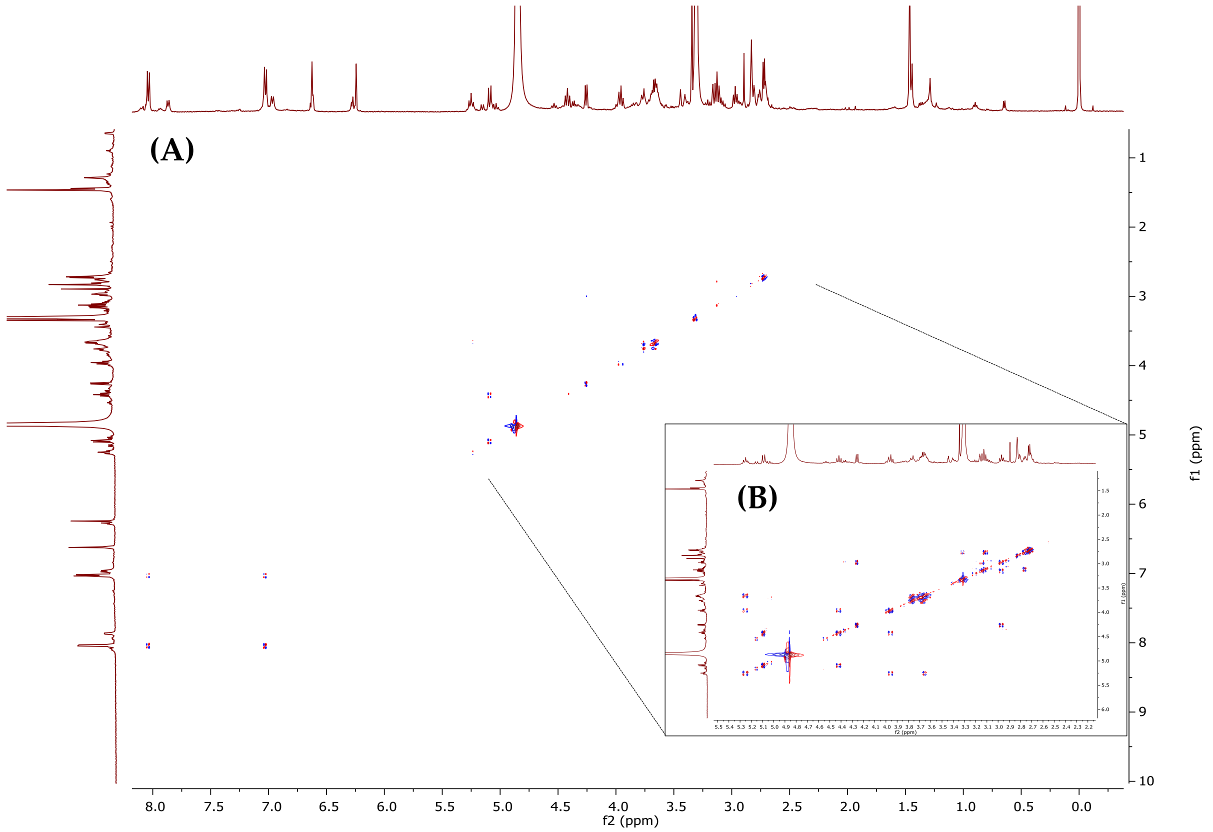

Figure S5: ^{1}H-^{1}H COSY spectrum of compound **1**; A) full spectrum, B) glycoside region (400 MHz, MeOH-*d*4)

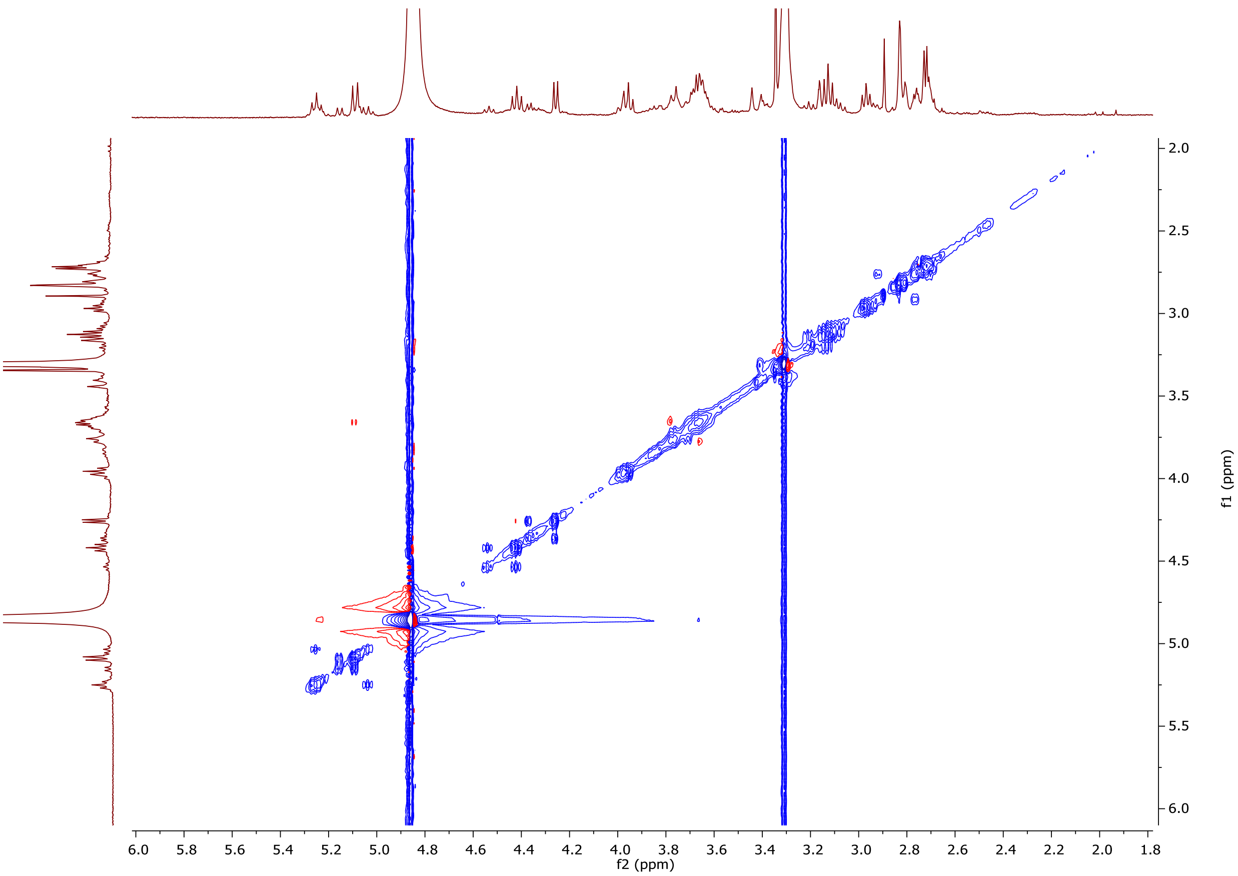

Figure S6: 2D-ROESY spectrum at glycoside region of compound **1** (400 MHz, MeOH-*d*4)

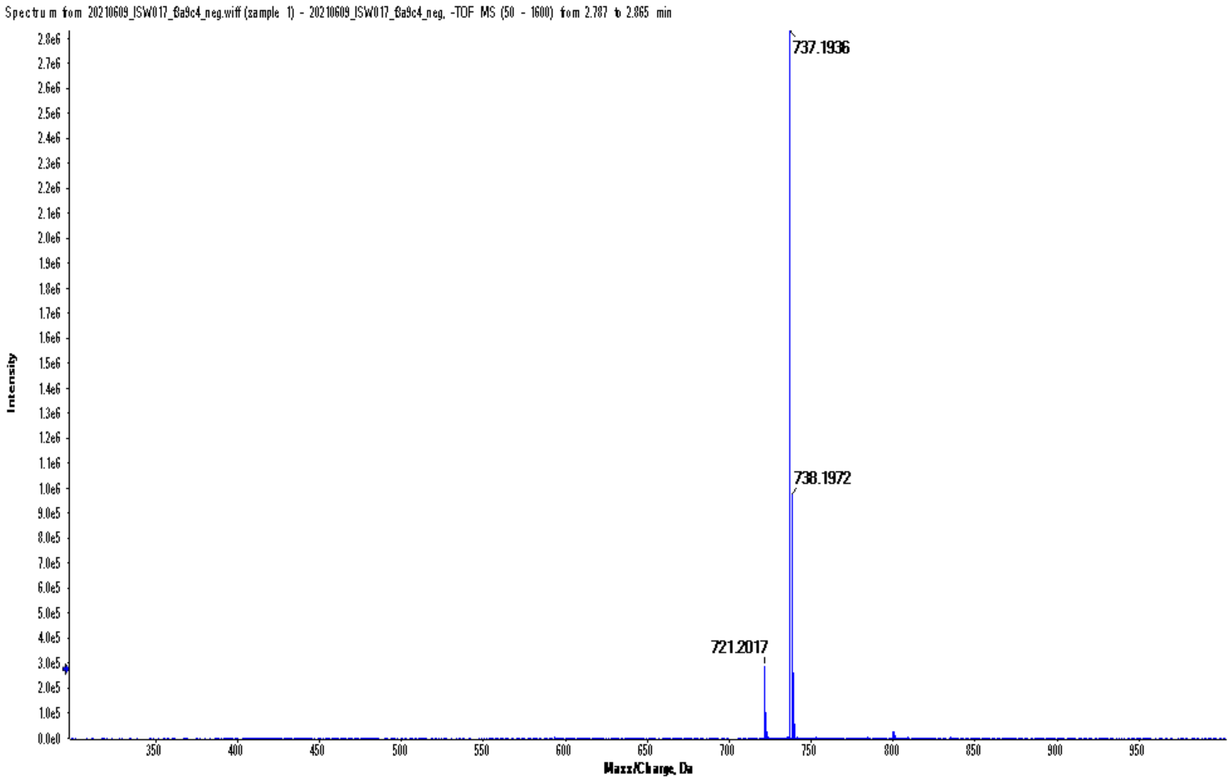

Figure S7: HR-ESI-MS spectrum of compound **1** in negative ion mode

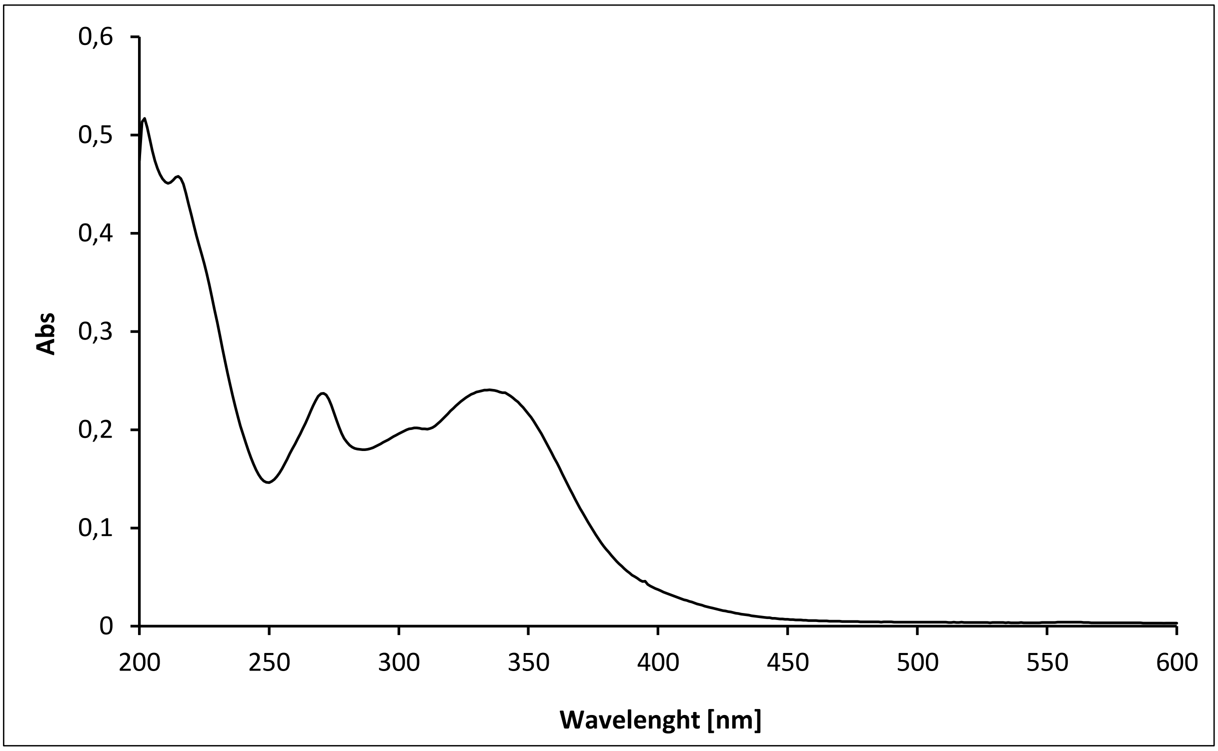

Figure S8: UV spectrum of compound **1** in MeOH

Table S1: Polarimeter data of compound **1**.

Light Source	WI
Wavelength	589 nm
Sample Aperture	Ø 3.0
Light Aperture	Ø 1.0
D.I.T.	5 sec
Cycle Times	20
Cycle Interval	5 sec
Path Length	50 mm
Concentration	0.153 W/V%
Factor	1.0000
Temp. Correct	0
**Sample**	**ISW017_f3a9c4**
Comment	1.53 mg / 1 ml Methanol
**Optical Rotation**	
Average	-0.0084 deg
S.D.	0.0003 deg
R.S.D.	4.1097 %
**Specific O.R.**	
Average	-10.9346
S.D.	0.4494
Temperatur	23.5° C

4-Allyl-5-methoxy-2-(*β*-D-glucopyranosyloxy-3-(4-methoxyphenyl)propanoate (Kadukoside)

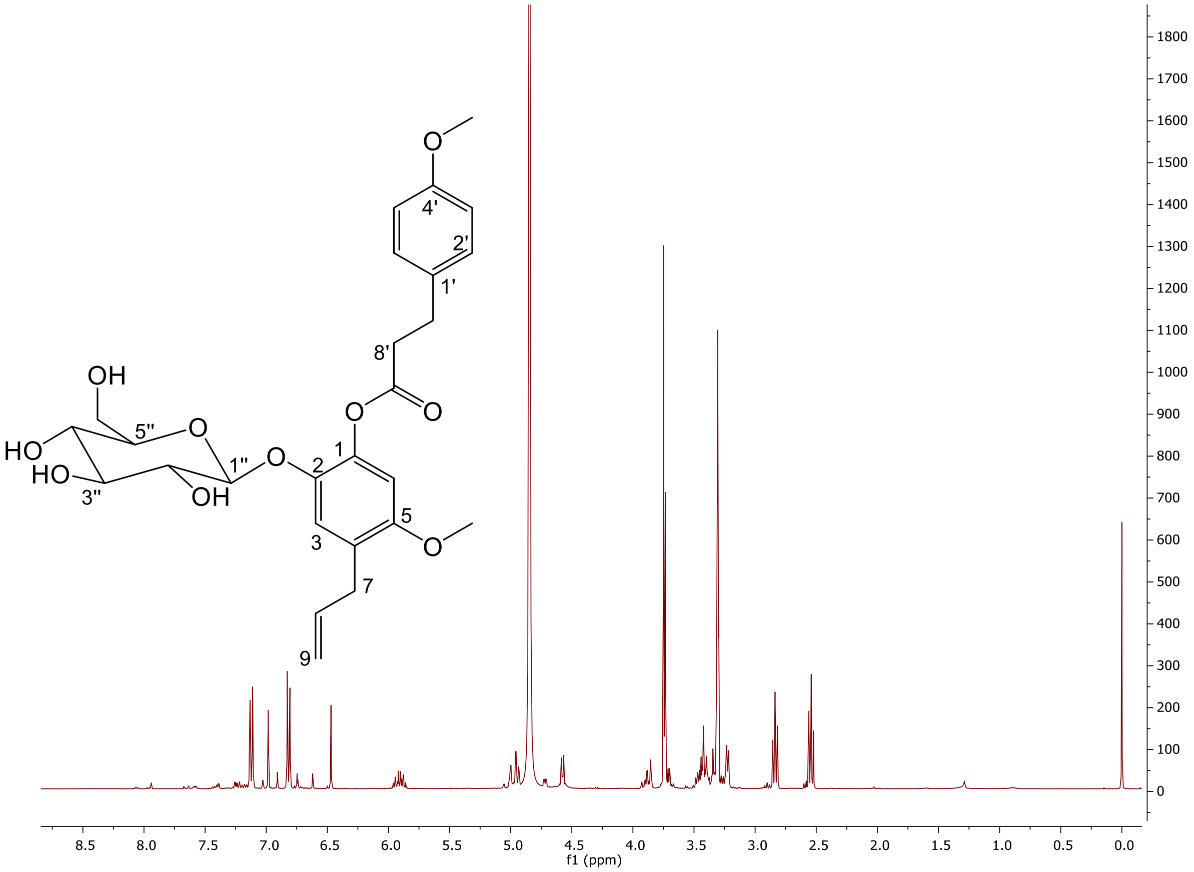

Figure S9: ^{1}H NMR spectrum of compound **2** (400 MHz, MeOH-*d*4)

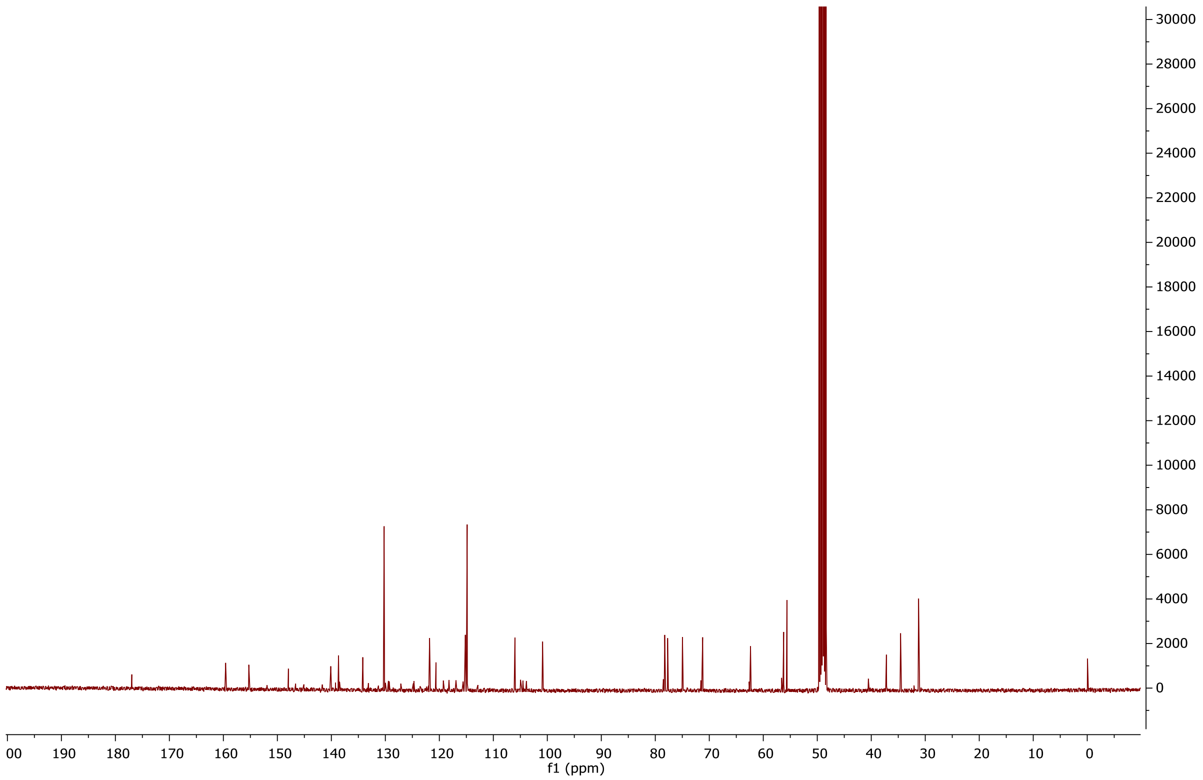

Figure S10 : ^{13}C NMR spectrum of compound **2** (400 MHz, MeOH-*d*4)

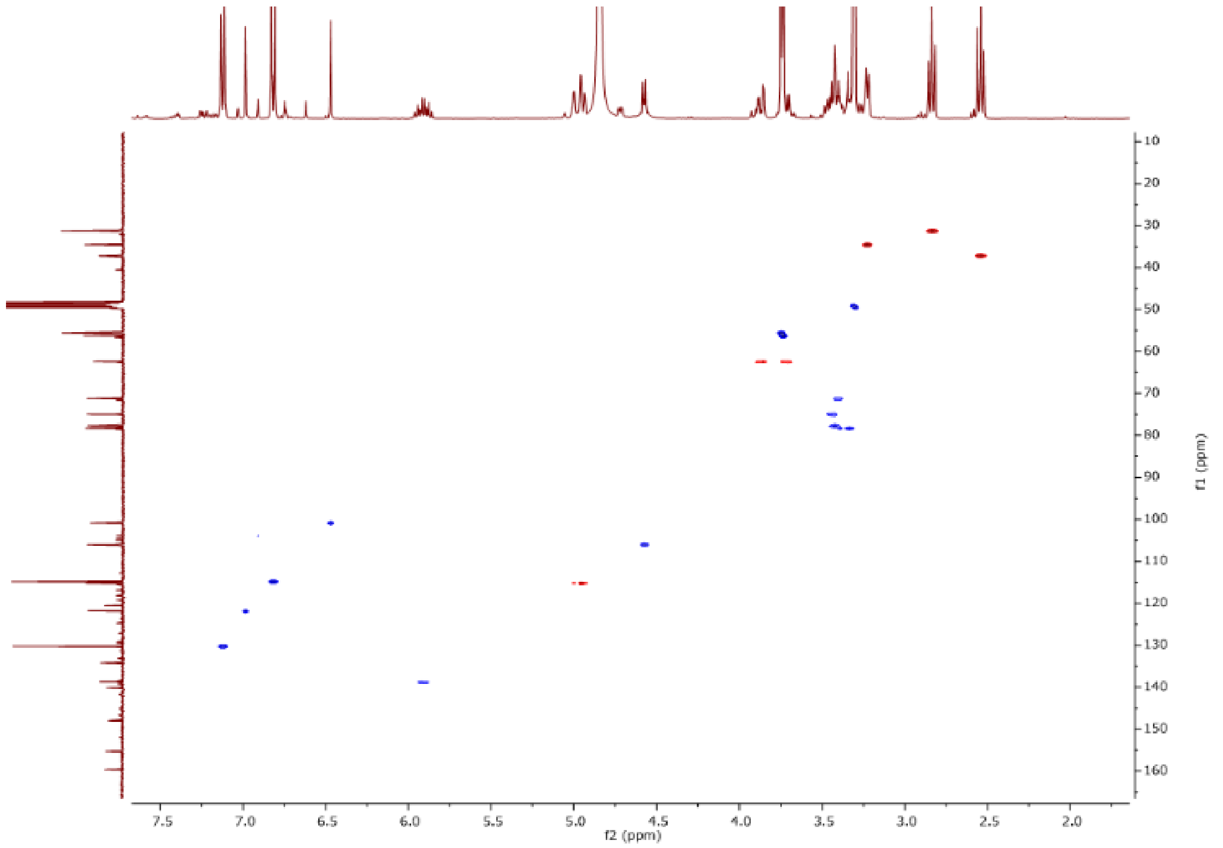

Figure S11: HSQC spectrum of compound **2** (400 MHz, MeOH-*d*4)

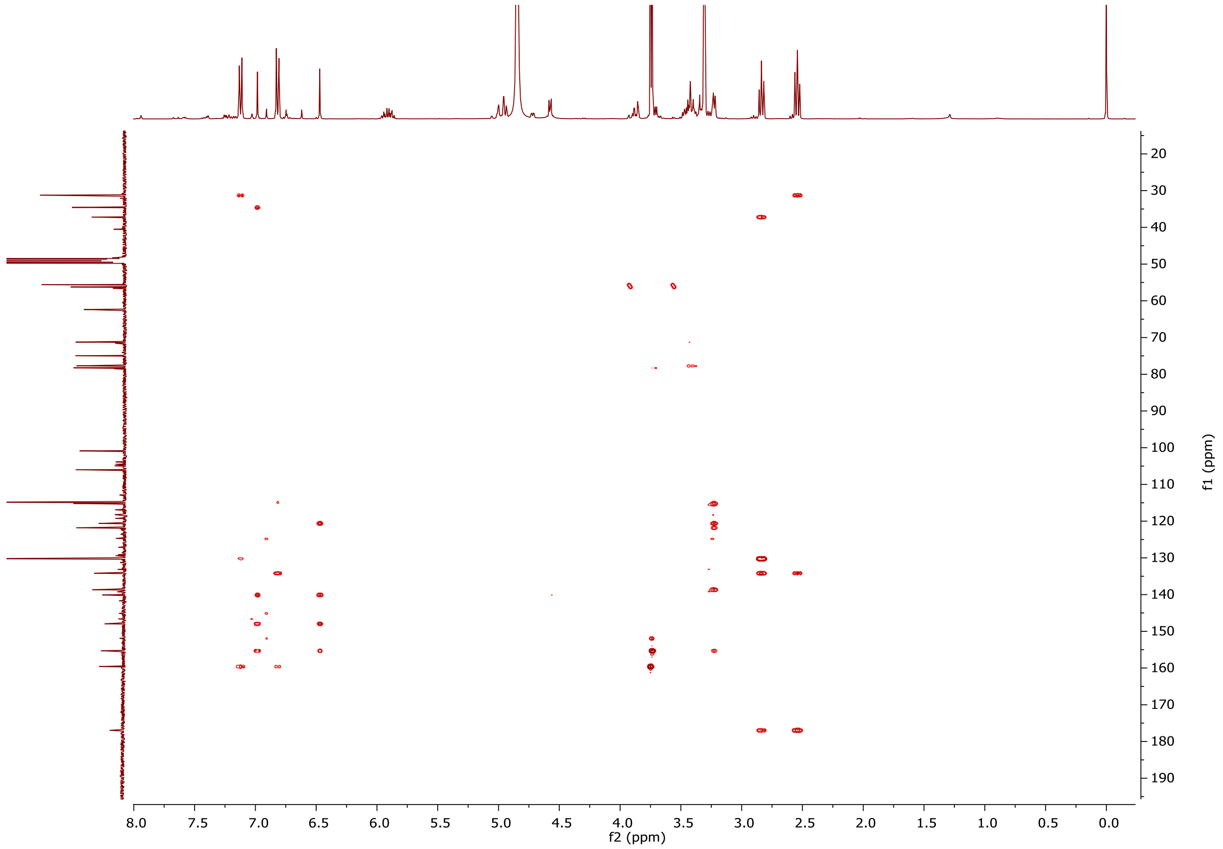

Figure S12: HMBC spectrum of compound **2** (400 MHz, MeOH-*d*4)

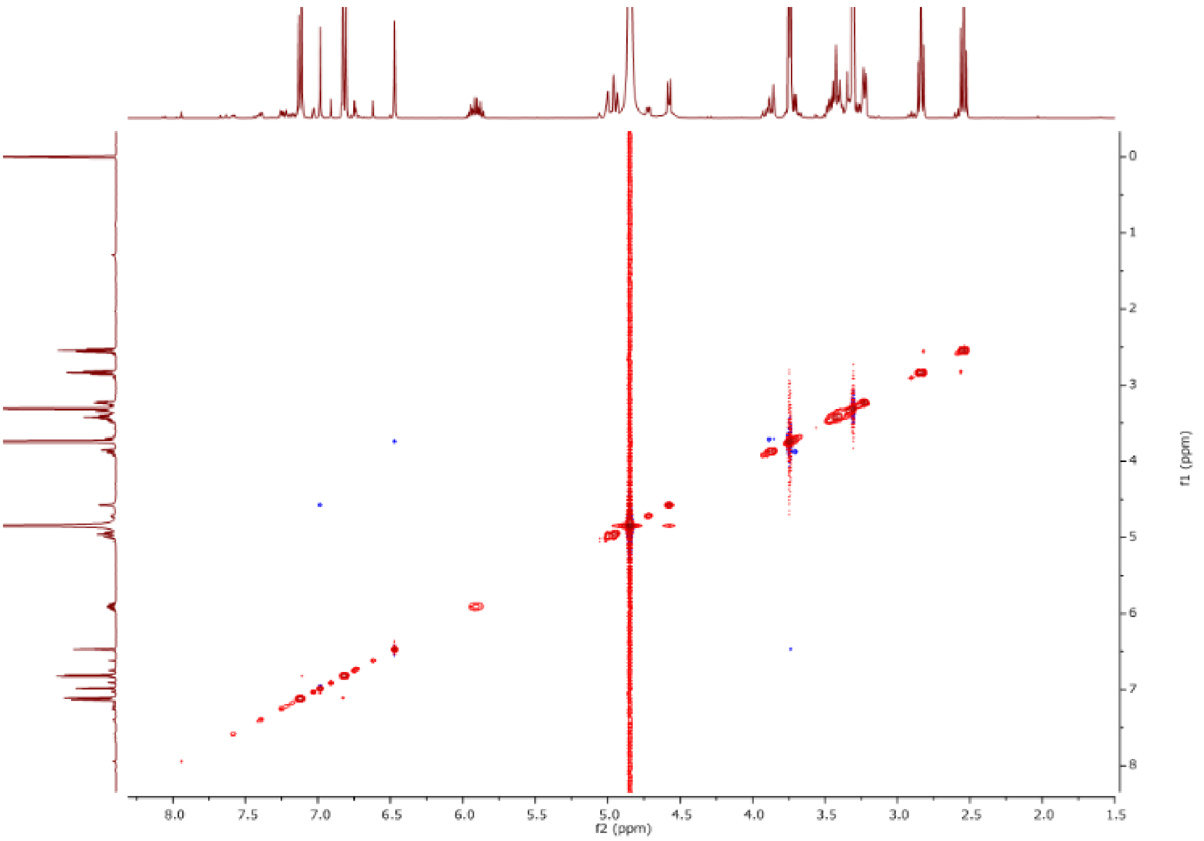

Figure S13: 2D-NOESY spectrum of compound **2** (400 MHz, MeOH-*d*4)

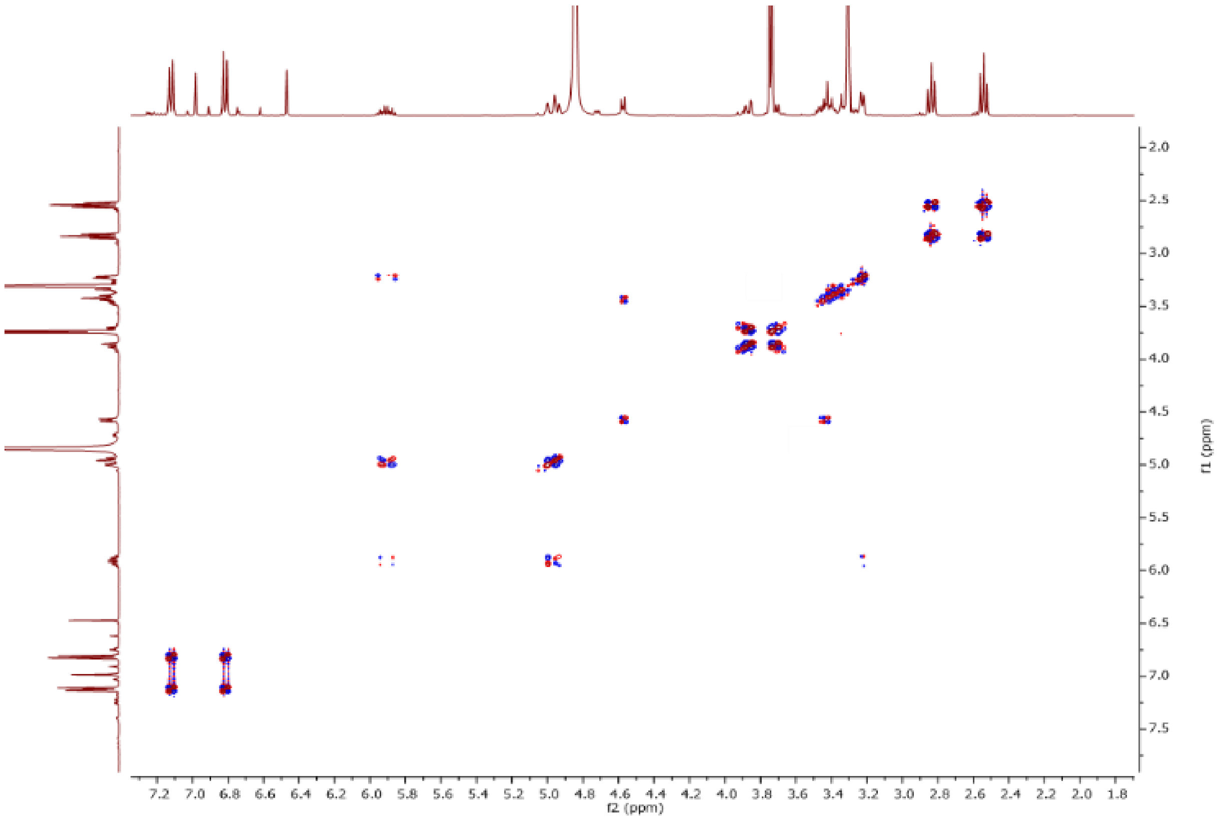

Figure S14: ^{1}H-^{1}H COSY spectrum of compound **2** (400 MHz, MeOH-*d*4)

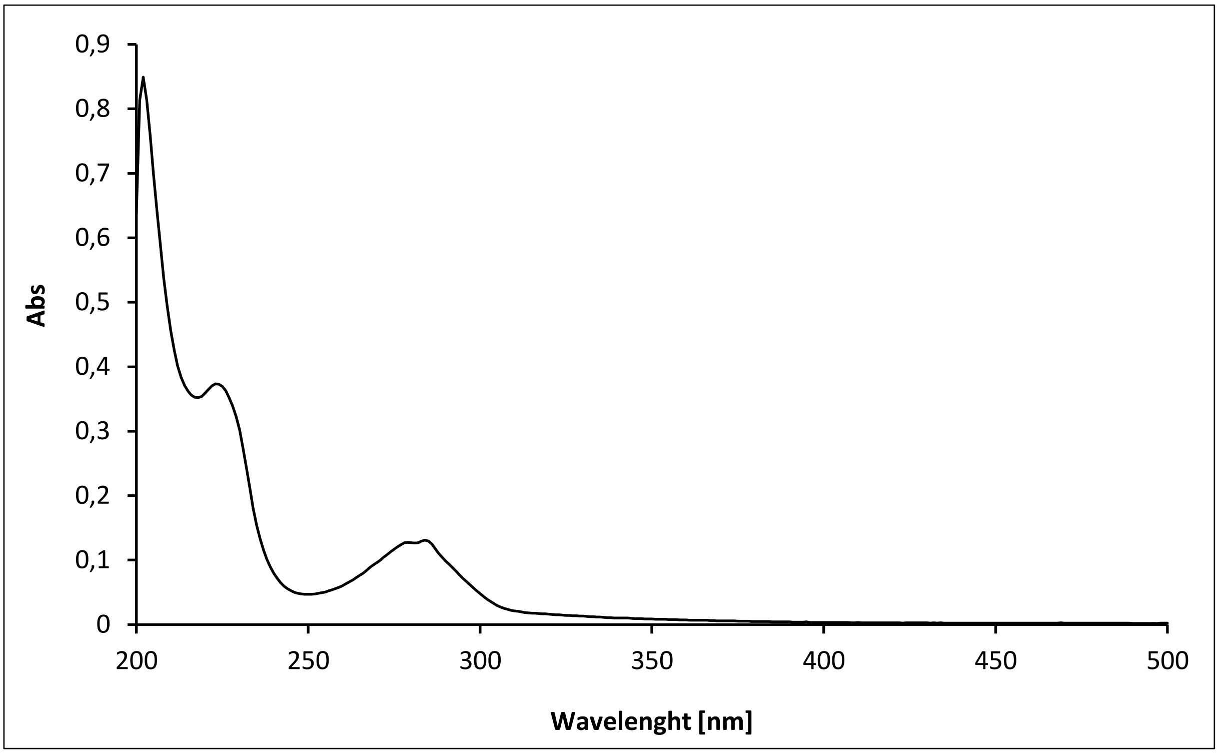

Figure S15: UV spectrum of compound **2** in MeOH

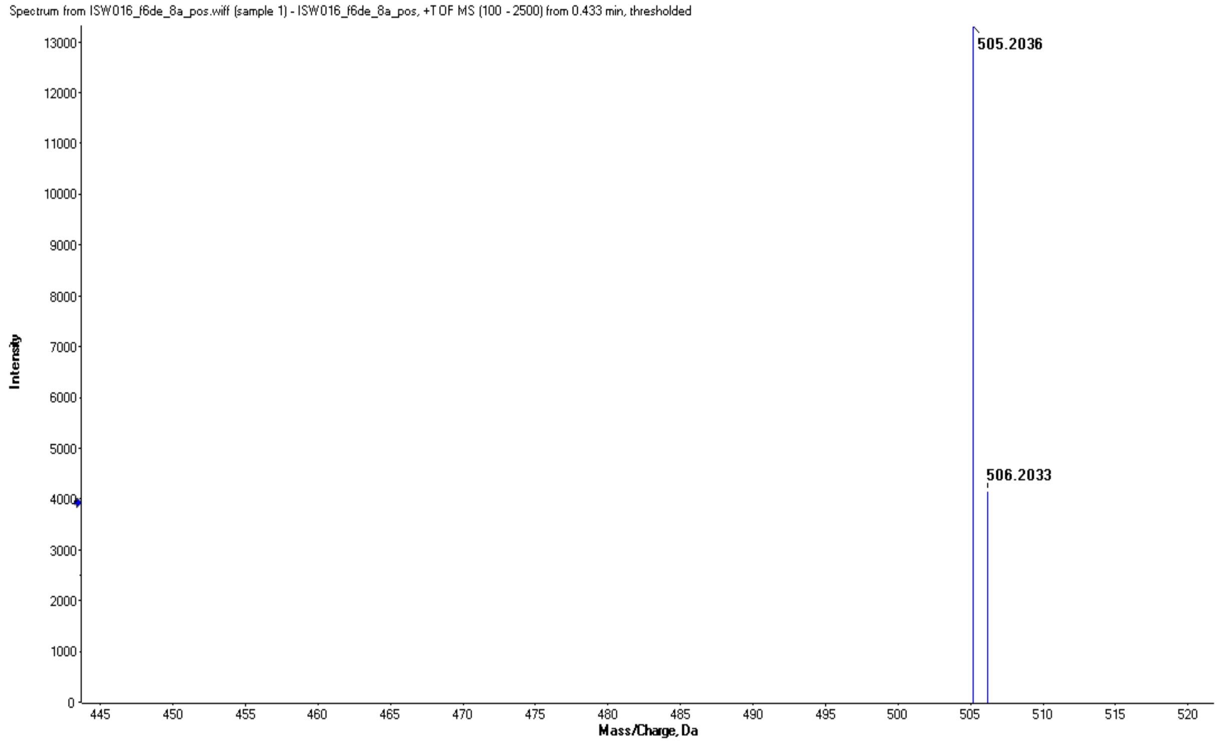

Figure S16: HR-ESI-MS spectrum of compound **2** in positive ion mode

6-*O*-*trans*-*p*-Coumaroyl-*D*-glucono-1,4-lactone

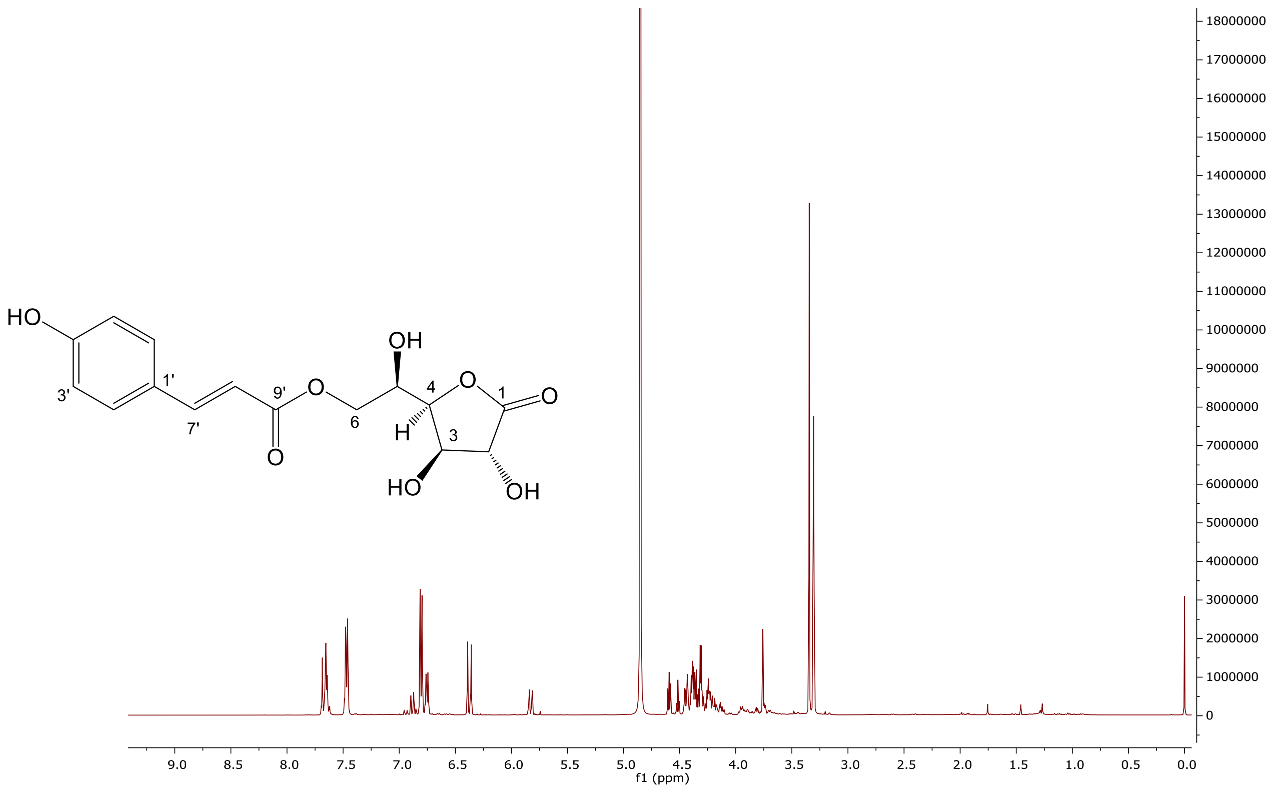

Figure S17: ^{1}H NMR spectrum of compound **3** (400 MHz, MeOH-*d*4)

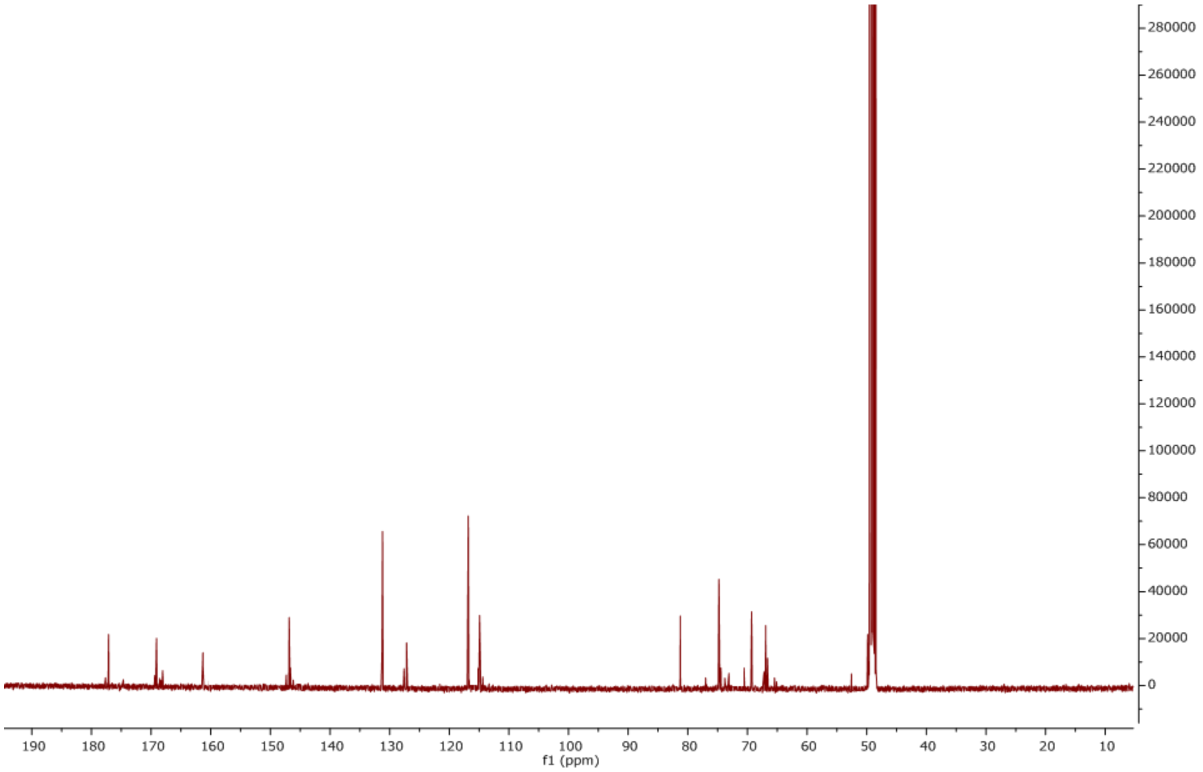

Figure S18: ^{13}C NMR spectrum of compound **3** (100 MHz, MeOH-*d*4)

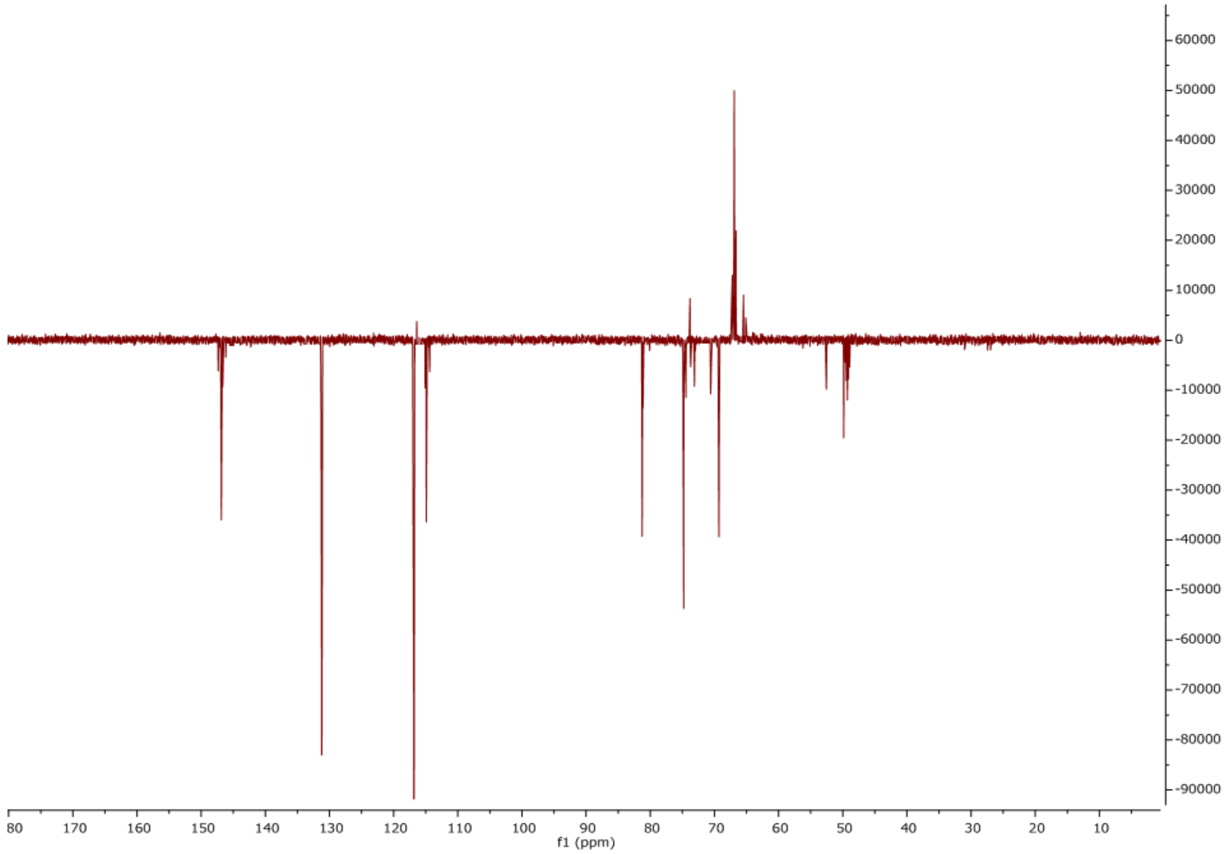

Figure S19: DEPT135 spectrum of compound **3** (100 MHz, MeOH-*d*4)

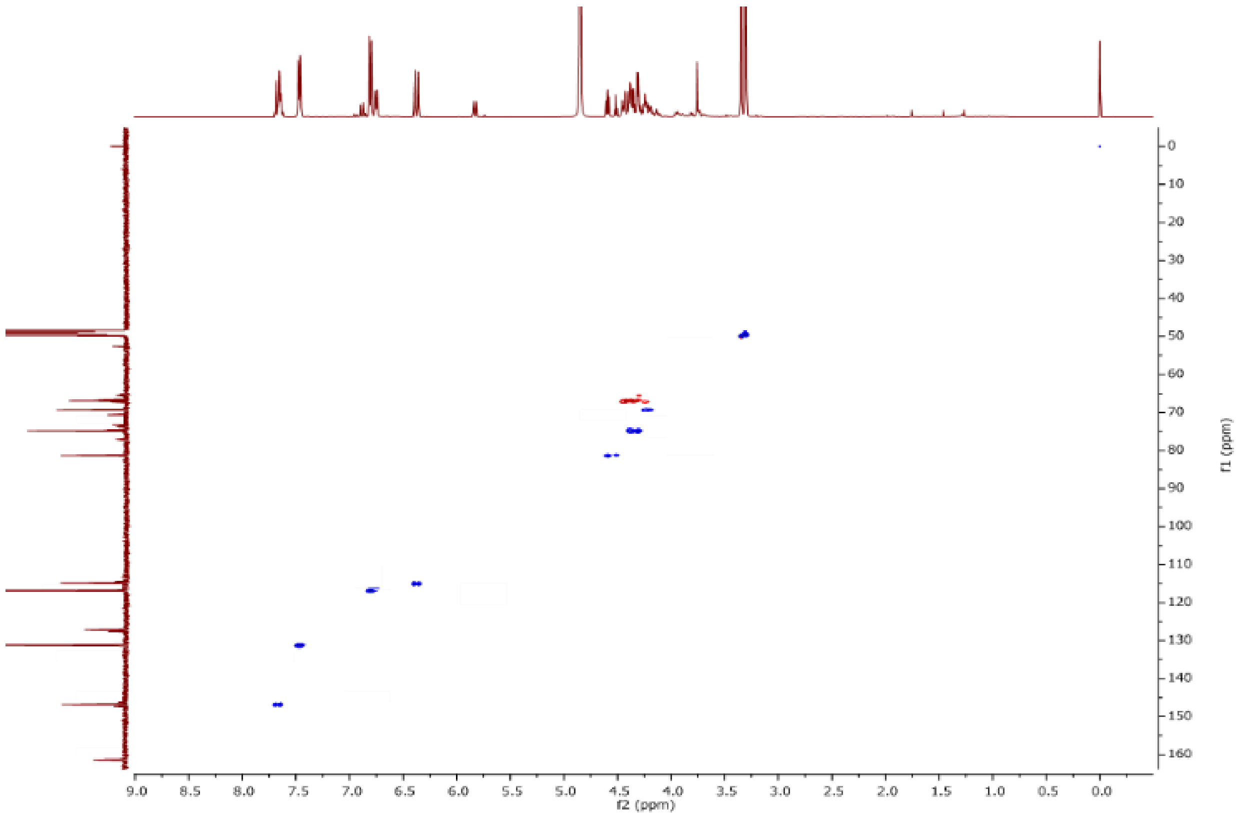

Figure S20: HSQC spectrum of compound **3** (400 MHz, MeOH-*d*4)

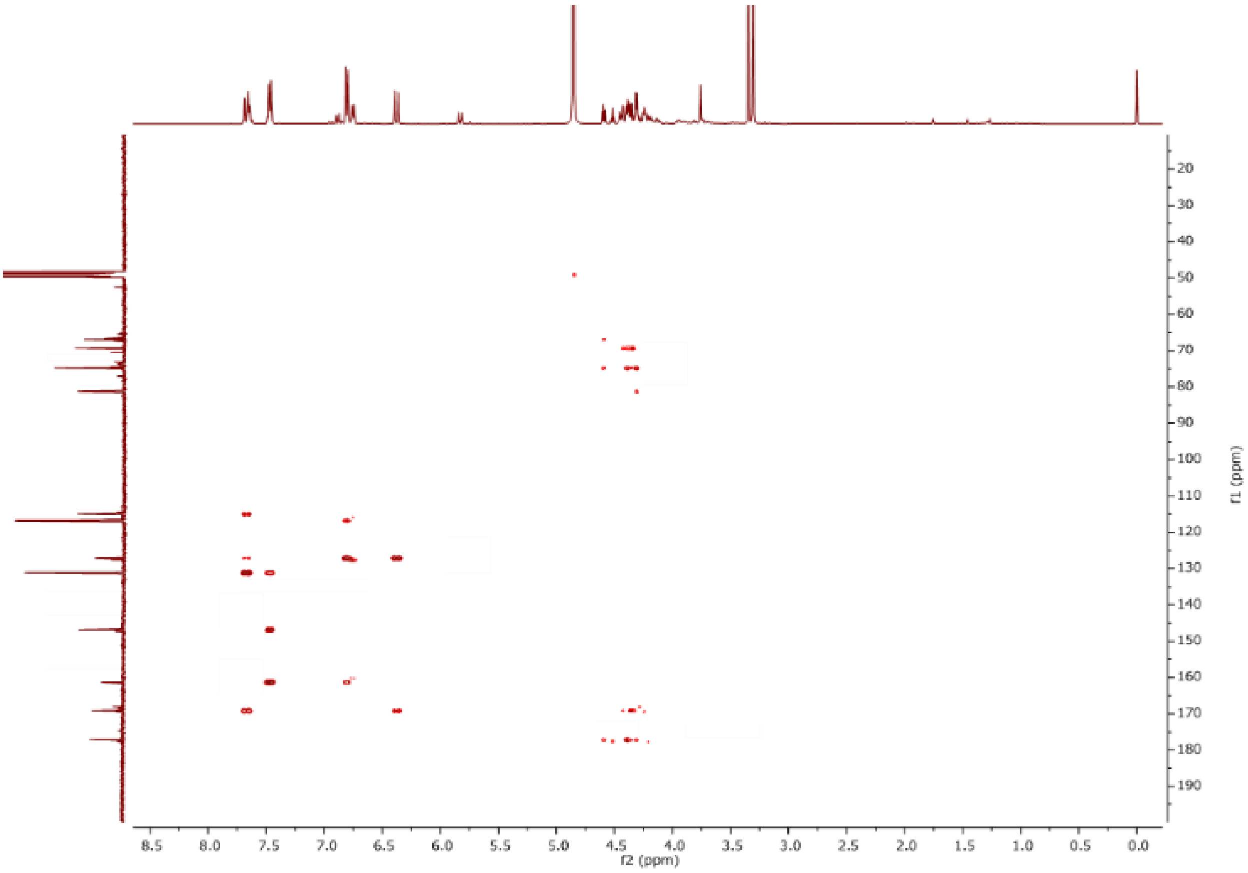

Figure S21: HMBC spectrum of compound **3** (400 MHz, MeOH-*d*4)

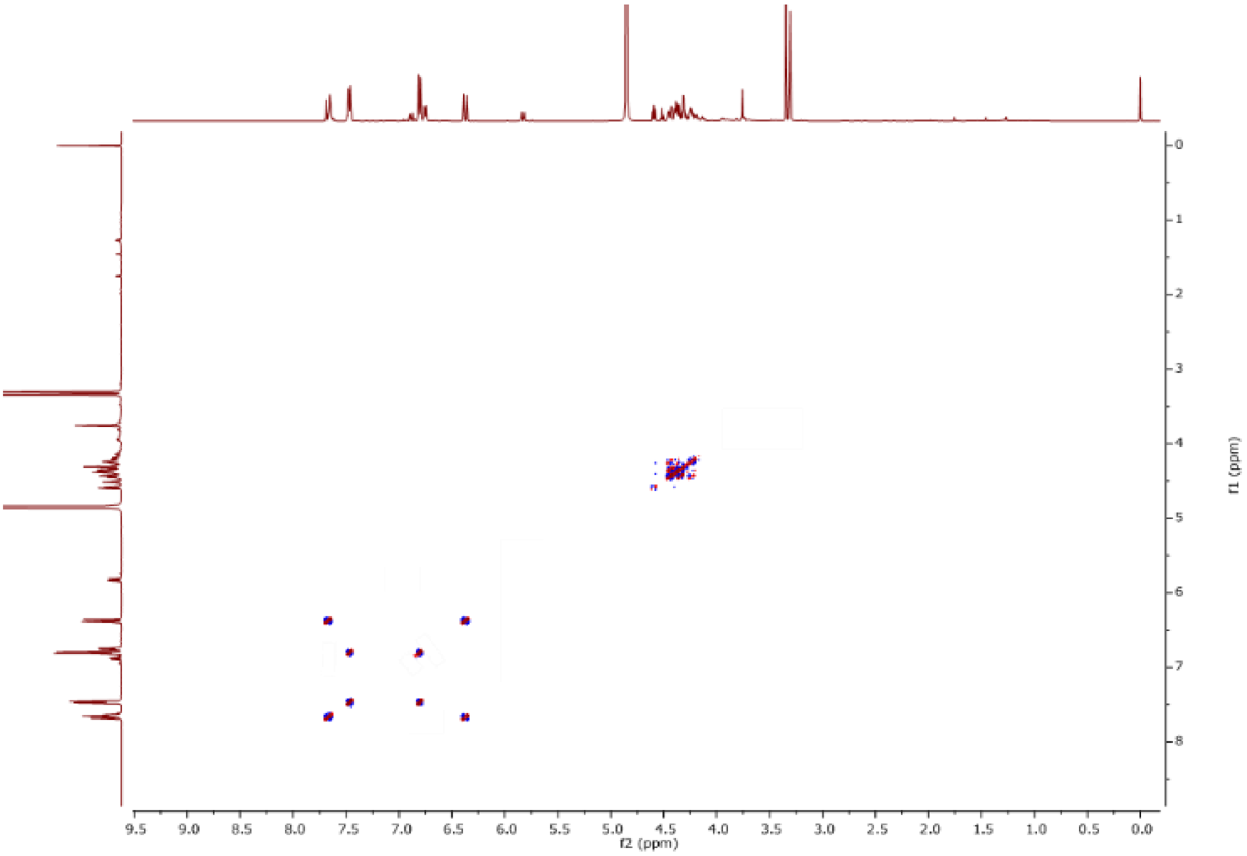

Figure S22: ^{1}H-^{1}H COSY spectrum of compound **3** (400 MHz, MeOH-*d*4)

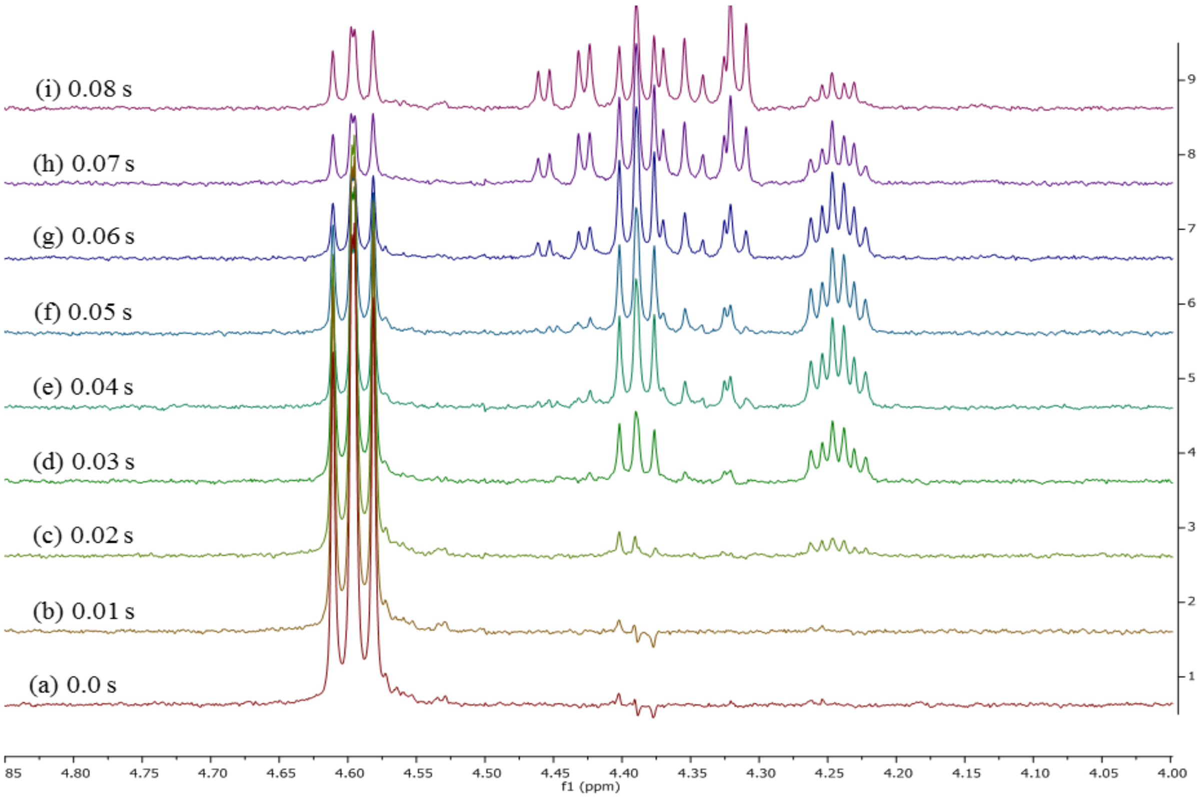

Figure S23: 1D-TOCSY spectrum of compound **3** with selective excitation of H-4 (δ_H 4.59) and mixing times (400 MHz, MeOH-*d*4)

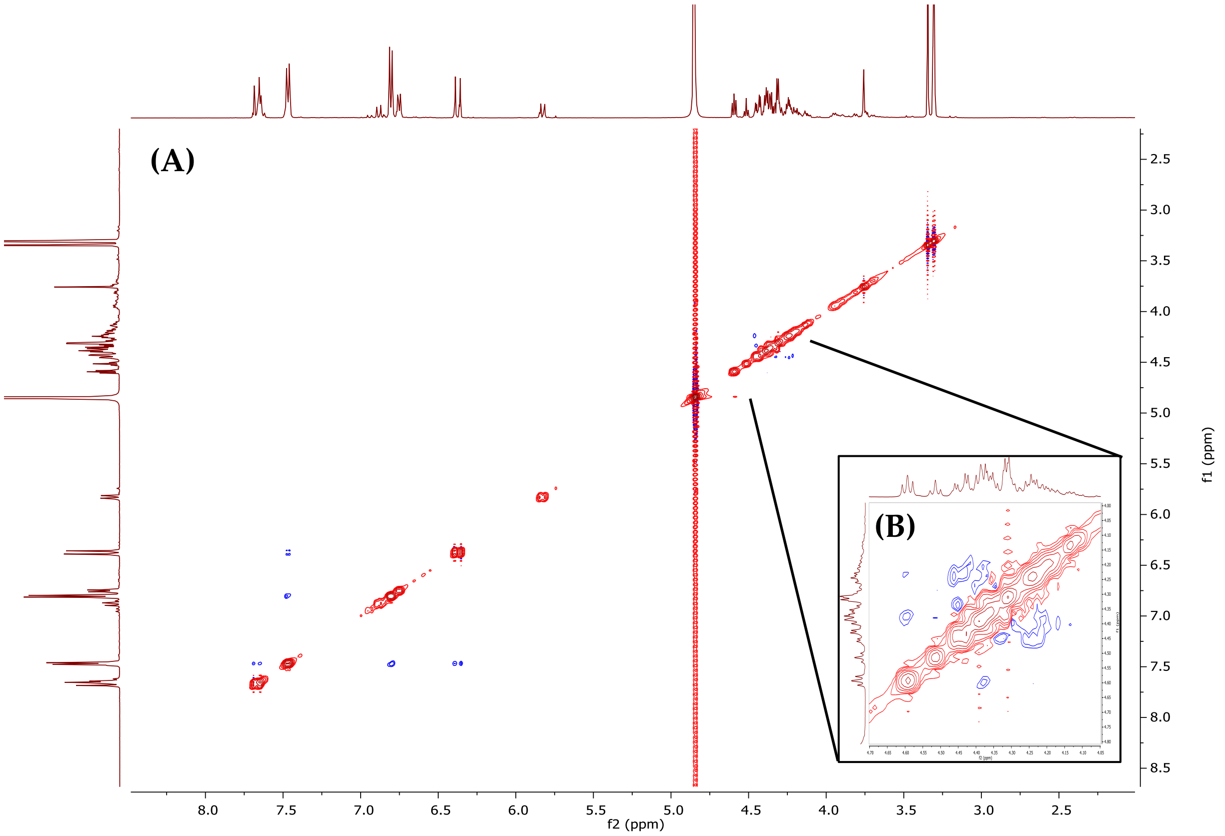

Figure S24: 2D-NOESY spectrum of compound **3;** A) full spectrum, B) glycoside region (400 MHz, MeOH-*d*4)

Table S2.^{1}H and ^{13}C NMR comparison data of compound **3** and similar structure (CD_3OD).

Pos.	Compound **3**		Tanaka et al. [37]	
	δ_H Mult. *J* (Hz) (400 MHz)	δ_C (100 MHz)	δ_H Mult. *J* (Hz) (600 MHz)	δ_C (150 MHz)
1		177.1		176.0
2	4.32 d 4.7	74.8	4.38 d 8.7	75.6
3	4.39 dd 5.4, 4.7	74.9	4.33 t 8.7	74.4
4	4.59 dd 6.3, 5.4	81.3	4.19 dd 8.7, 2.4	81.4
5	4.24 ddd 6.3, 6.3, 3.3	69.3	4.07 m	67.8
6A	4.35 dd 11.7, 3.3	66.9	4.29 dd 11.1, 6.0	65.9
6B	4.44dd 11.7, 6.3	66.9	4.33 dd 11.1, 6.9	65.9
1′		127.2		127.1
2′	7.47 d 8.7	131.2	7.47 d 9.0	131.2
3′	6.81 d 8.7	116.8	6.81 d 9.0	116.8
4′		161.3		161.4
5′	6.81 d 8.7	116.8	6.81 d 9.0	116.8
6′	7.47 d 8.7	131.2	7.47 d 9.0	131.2
7′	7.67 d 16.0	146.9	7.67 d 15.9	147.0
8′	6.38 d 16.0	114.9	6.37 d 15.9	114.8
9′		169.1		168.9

[37] Tanaka, Y.; Yanagida, A.; Komeya, S.; Kawana, M.; Honma, D.; Tagashira, M.; Kanda, T.; Shibusawa, Y. Comprehensive separation and structural analyses of polyphenols and related compounds from bracts of hops (*Humulus lupulus* L.). *J Agric. Food Chem.* **2014**, 62, 2198-2206.

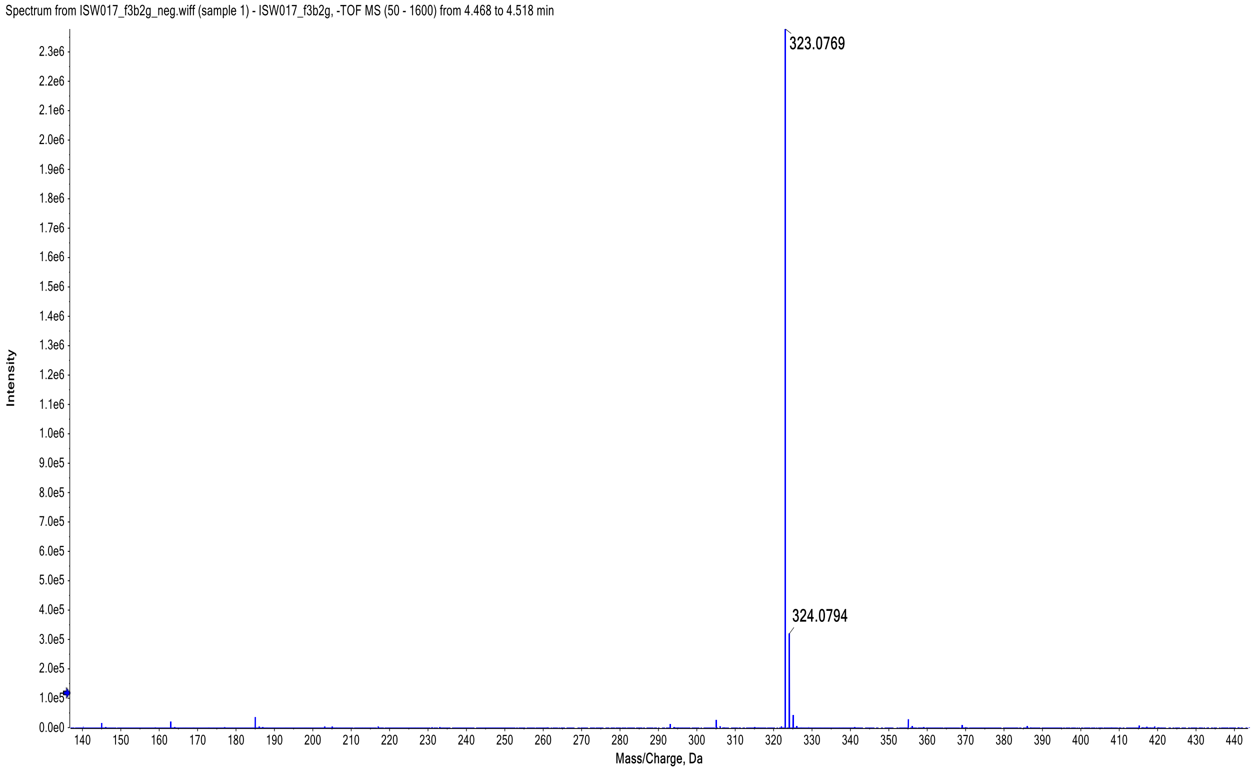

Figure S25: HR-ESI-MS spectrum of compound **3** in negative ion mode

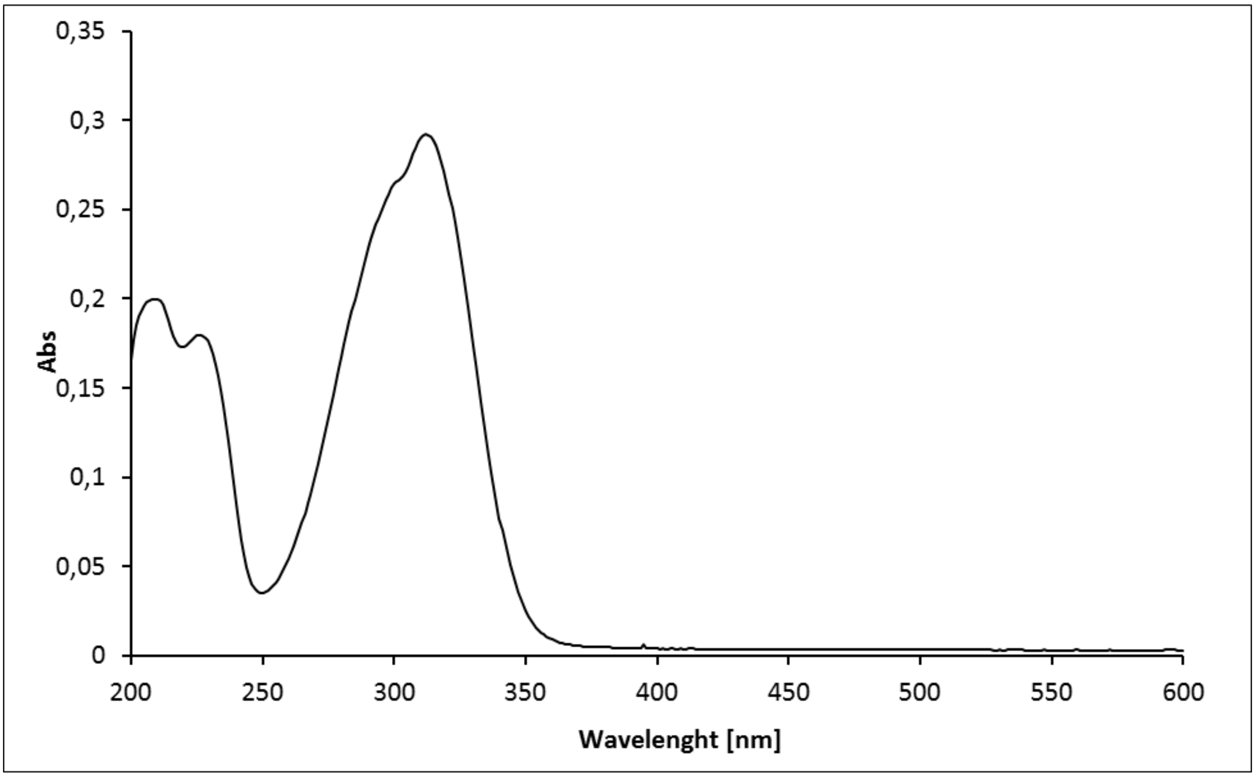

Figure S26: UV spectrum of compound **3** in MeOH

Table S3: Polarimeter data of compound **3**.

Light Source	WI
Wavelength	589 nm
Sample Aperture	Ø 3.0
Light Aperture	Ø 1.0
D.I.T.	5 sec
Cycle Times	20
Cycle Interval	5 sec
Path Length	50 mm
Concentration	0.247 W/V%
Factor	1.0000
Temp. Correct	0
**Sample**	**ISW017_f3b2g**
Comment	2.47 mg / 1 ml Methanol
**Optical Rotation**	
Average	0.0050 deg
S.D.	0.0002 deg
R.S.D.	4.2827 %
**Specific O.R.**	
Average	4.0445
S.D.	0.1732
Temperatur	23.2° C

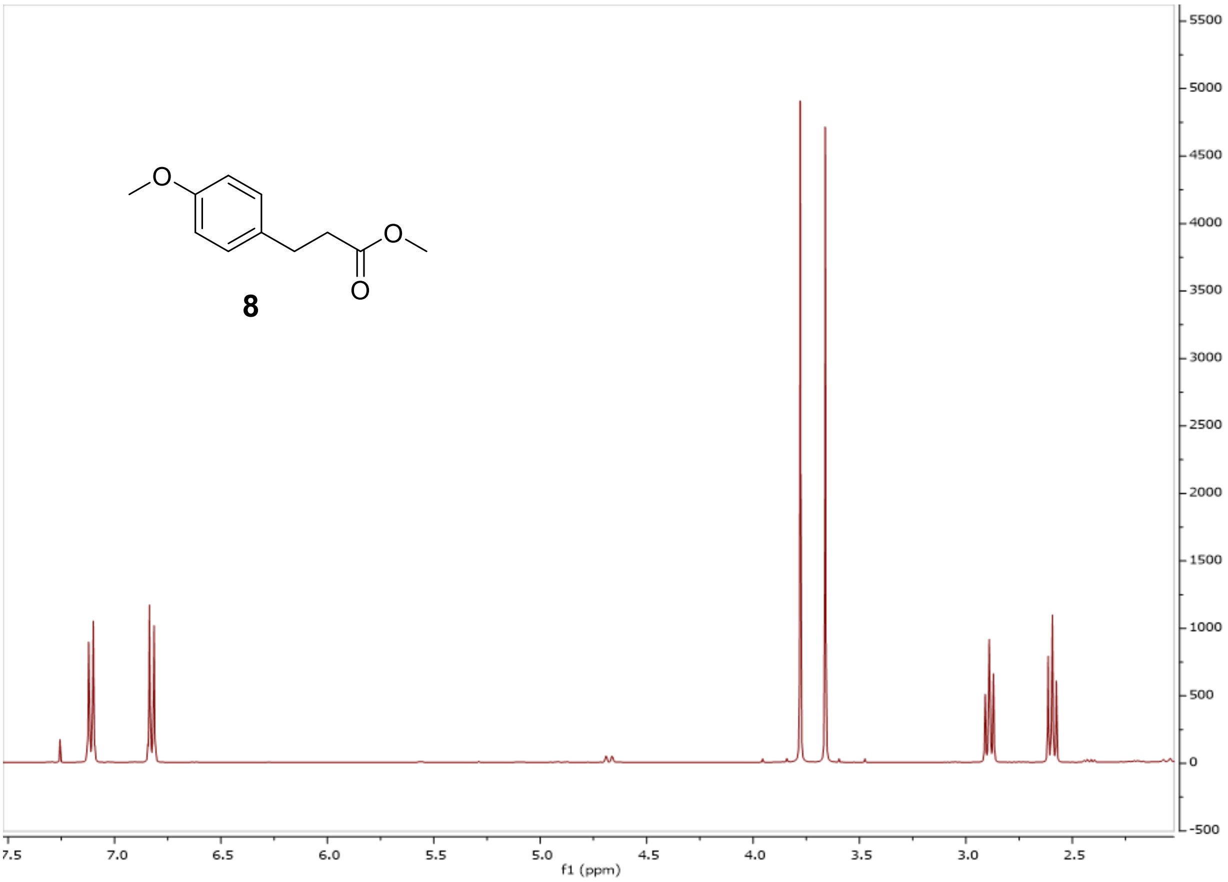

Figure S27: ^{1}H NMR spectrum of compound **8** (400 MHz, $CDCl_3$)

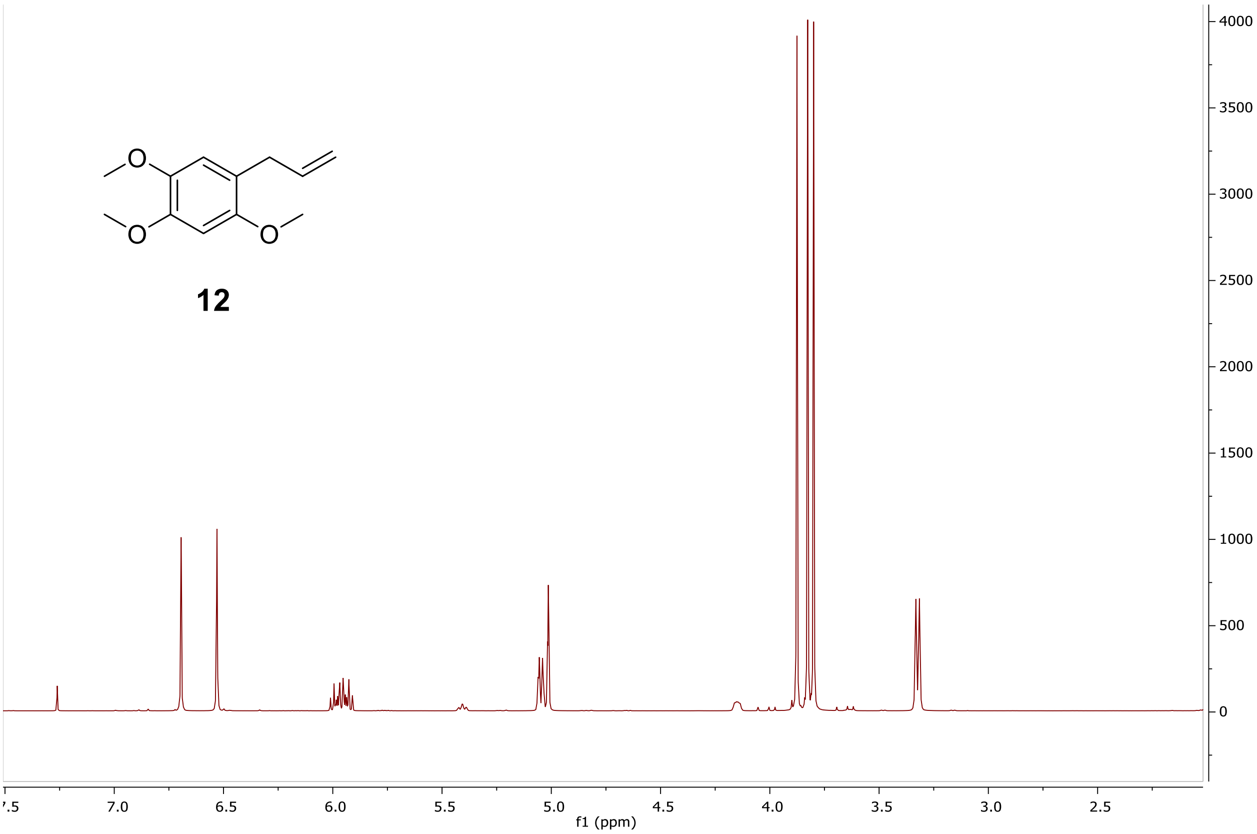

Figure S28: ^{1}H NMR spectrum of compound **12** (400 MHz, $CDCl_3$)

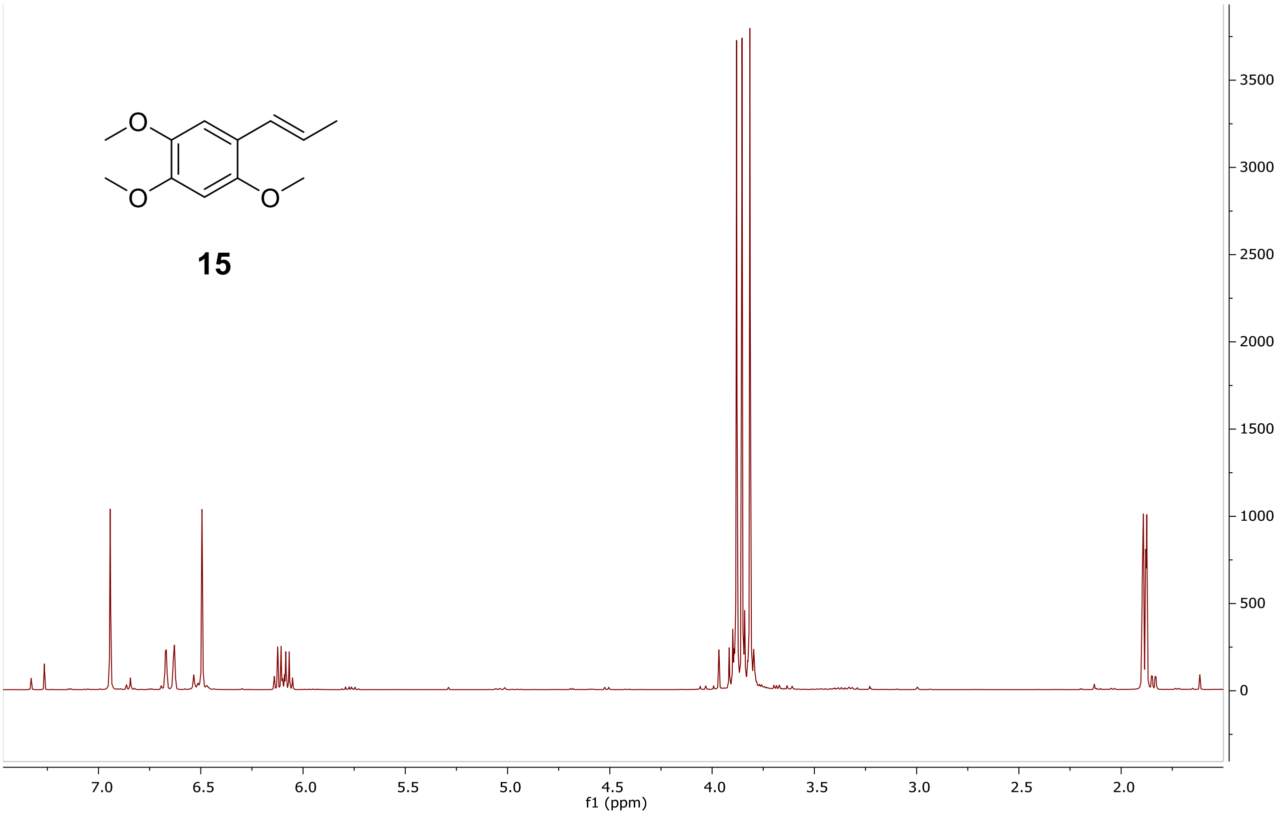

Figure S29: ^{1}H NMR spectrum of compound **15** (400 MHz, $CDCl_3$)

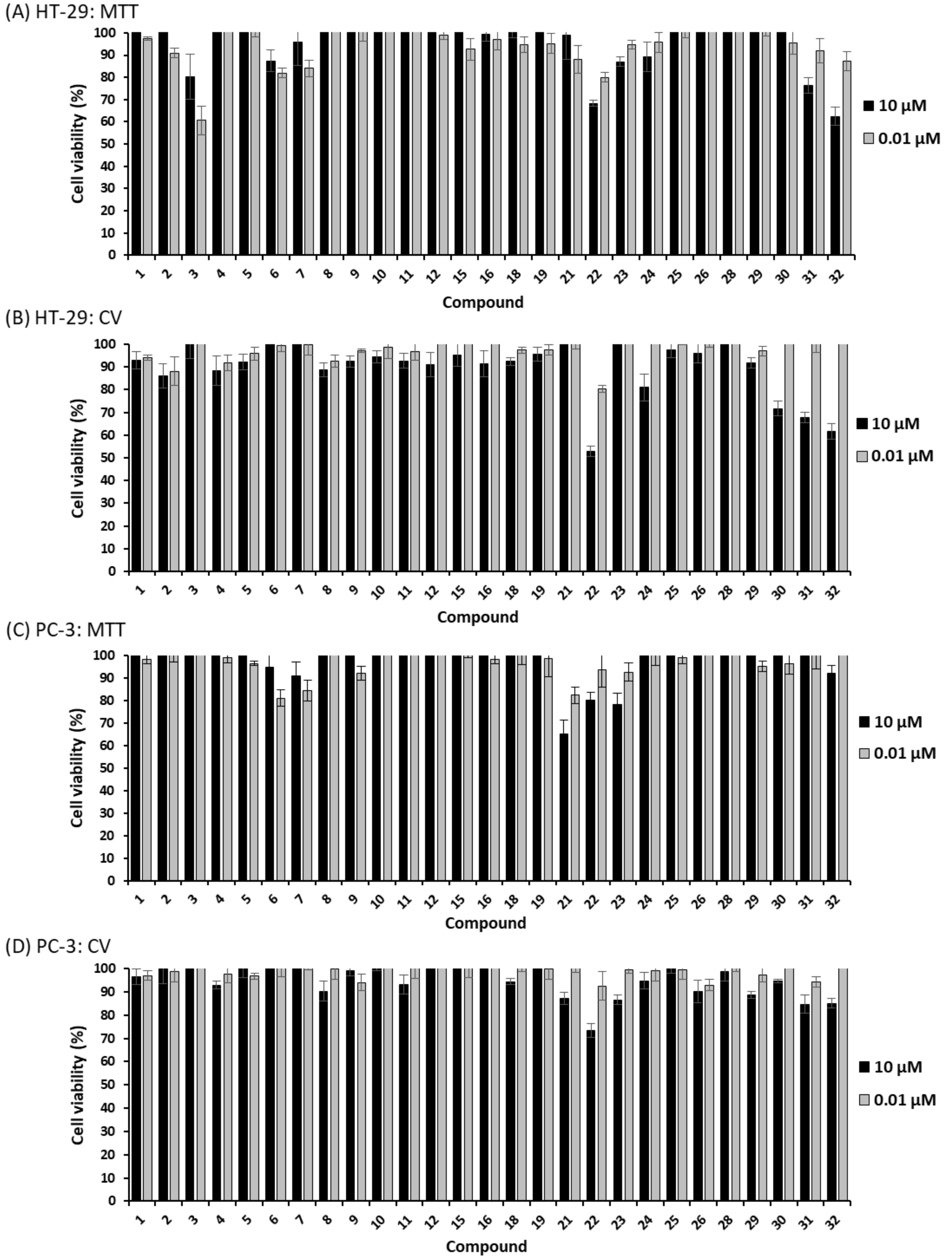

Figure S30: Antiproliferative and cytotoxic activities (MTT and CV) of compounds isolated from *P. sarmentosum* against human colorectal (HT-29) and prostate (PC-3) cancer cell lines. Digitonin (125 µM) was used as positive control compromising the cells to the point of 0% of cell viability after 48 h (data not shown).
